# Variability of the Human Serum Metabolome over 3 Months in the EXPOsOMICS Personal Exposure Monitoring Study

**DOI:** 10.1021/acs.est.3c03233

**Published:** 2023-08-15

**Authors:** Max J. Oosterwegel, Dorina Ibi, Lützen Portengen, Nicole Probst-Hensch, Sonia Tarallo, Alessio Naccarati, Medea Imboden, Ayoung Jeong, Nivonirina Robinot, Augustin Scalbert, Andre F. S. Amaral, Erik van Nunen, John Gulliver, Marc Chadeau-Hyam, Paolo Vineis, Roel Vermeulen, Pekka Keski-Rahkonen, Jelle Vlaanderen

**Affiliations:** † Division of Environmental Epidemiology, Institute for Risk Assessment Sciences, Utrecht University, Utrecht 3584 CM, The Netherlands; ‡ Julius Center for Health Sciences and Primary Care, University Medical Center Utrecht, Utrecht 3508 GA, The Netherlands; § Medical Research Council-Public Health England Center for Environment and Health, Department of Epidemiology and Biostatistics, Imperial College London, London SW7 2AZ, U.K.; ∥ Swiss Tropical and Public Health Institute, Allschwil 4123, Switzerland; ⊥ University of Basel, Basel 4001, Switzerland; # Italian Institute for Genomic Medicine (IIGM), c/o IRCCS, Turin 10060, Italy; ∇ National Heart and Lung Institute, Imperial College London, London SW3 6LY, U.K.; ○ Nutrition and Metabolism Branch, International Agency for Research on Cancer, World Health Organization, Lyon CS 90627, France; ◆ Centre for Environmental Health and Sustainability & School of Geography, Geology and the Environment, University of Leicester, Leicester LE1 7RH, U.K.; ¶ NIHR Imperial Biomedical Research Centre, London W2 1NY, U.K.

**Keywords:** blood, biomarkers, metabolomics, repeatability, variability, liquid chromatography coupled to high-resolution mass spectrometry (LC-HRMS), epidemiology, cohort study, reliability, intraclass correlation coefficient (ICC), within-individual variability, between-individual variability

## Abstract

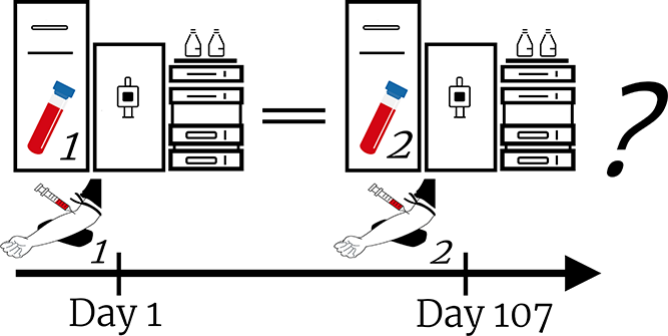

Liquid chromatography coupled to high-resolution mass spectrometry (LC-HRMS) and untargeted metabolomics are increasingly used in exposome studies to study the interactions between nongenetic factors and the blood metabolome. To reliably and efficiently link detected compounds to exposures and health phenotypes in such studies, it is important to understand the variability in metabolome measures. We assessed the within- and between-subject variability of untargeted LC-HRMS measurements in 298 nonfasting human serum samples collected on two occasions from 157 subjects. Samples were collected ca. 107 (IQR: 34) days apart as part of the multicenter EXPOsOMICS Personal Exposure Monitoring study. In total, 4294 metabolic features were detected, and 184 unique compounds could be identified with high confidence. The median intraclass correlation coefficient (ICC) across all metabolic features was 0.51 (IQR: 0.29) and 0.64 (IQR: 0.25) for the 184 uniquely identified compounds. For this group, the median ICC marginally changed (0.63) when we included common confounders (age, sex, and body mass index) in the regression model. When grouping compounds by compound class, the ICC was largest among glycerophospholipids (median ICC 0.70) and steroids (0.67), and lowest for amino acids (0.61) and the O-acylcarnitine class (0.44). ICCs varied substantially within chemical classes. Our results suggest that the metabolome as measured with untargeted LC-HRMS is fairly stable (ICC > 0.5) over 100 days for more than half of the features monitored in our study, to reflect average levels across this time period. Variance across the metabolome will result in differential measurement error across the metabolome, which needs to be considered in the interpretation of metabolome results.

## Abstract 2

Limited insight exists on the repeatability of untargeted metabolomic measurements of human serum samples. This study estimates its repeatability over 100 days with implications for exposome research.

## Introduction

Untargeted metabolomics techniques are increasingly used in epidemiological studies of chronic diseases (e.g.,^1,2^) and the exposome.^3,4^ Untargeted liquid chromatography coupled to high-resolution mass spectrometry (LC-HRMS) provides an efficient means for broad-scale assessment of the metabolome by measuring thousands of the metabolome by measuring thousands of endogenous and exogenous compounds as well as their transformation products.^5^

Correct interpretation of untargeted LC-HRMS data in metabolome studies requires insight into the variability of the measurements. This is especially relevant for studies where repeated samples are not available, and measurements are done in a single biological sample. The implicit assumption is that the measured features reasonably reflect longer-term average levels. For metabolites that express substantial temporal variation or those for which the assay precision is low, a single measurement may be a poor reflection of their usual levels over a time period. This can bias the exposome–metabolome or metabolome–disease associations and reduce the power of the study.^6^

To maximize the efficiency of a cohort study, most of the variability in metabolite measurements needs to be between individuals and not attributable to the analytical measurement error and short-term intraindividual changes irrelevant to the long-term biological state. Only interindividual variability encompasses measurable differences that can be associated with the chronic disease of interest. This concept can be quantified by the intraclass correlation coefficient (ICC) as the proportion of the total variance (consisting of within- and between-subject) explained by between-subject variance, ranging from 0 to 1.^7,8^ Under a “single sample per person in a cohort study, or nested case–control, design” a higher ICC is favorable.^6^

Previous work found reasonable variability (median ICCs ranging from 0.50 to 0.60) of the blood metabolome on the short (several weeks) to medium (several months) term based on LC-MS measurements.^9−15^ Most of these studies used targeted measurement methods. The number of studies evaluating the variability of the blood metabolome using untargeted LC-HRMS is limited.^10,12^ In addition, existing work did not explicitly model the censoring of the metabolite levels. Excluding nondetects or a flawed imputation method like simple substitution (with some fraction of the detection limit) can bias the variance components.^16^

## Methods

### Study Population

The design of the EXPOsOMICS PEM study has been described before.^17−20^ In brief, 166 individuals were recruited in four European areas: Utrecht and Amsterdam (referred to as Utrecht hereafter), Turin, Norwich, and Basel. Subjects were excluded if they smoked or lived with a smoker or ex-smoker (quit less than 6 months ago), were younger than 50 years or older than 70 years of age at the start of the study, used doctor-prescribed medication, were restricted in daily activities due to physical limitations, or if the individual had moved much closer to a busy road (or vice versa) since original cohort inclusion. Additionally, subjects were excluded if they had a job that involved contact with major occupational chemical exposures such as diesel exhaust, or had a doctor-diagnosed chronic disease such as ischemic heart disease, cardiovascular disease, chronic obstructive pulmonary disease, asthma, diabetes, or a nonmelanoma skin cancer. Approximately half of the recruited individuals lived on a major road, while the other half-lived at least 100 m away from such a road. A major road was defined as a road with >10 000 cars per day or a street canyon with more than 5000 cars/day. The study was conducted from December 2013 to September 2015. Participants performed their own daily routine during the three personal exposure monitoring sessions, in different seasons over the span of one year.

After each session, nonfasting blood samples were collected in a seated position from the participant by a nurse. Blood was taken by standard phlebotomy technique of venipuncture of a forearm vein. In Turin, the blood was collected in a clinic in the afternoon, while in the other cities, the blood was drawn at the participants’ home in the morning. Blood samples were stored in −80 °C freezers within 2 h of collection. During transport to the freezer, the samples were stored in a cooling bag or box. The serum fraction was prepared by centrifugation of the blood collection tube at 2500 g for 15 min at 4 °C. The serum of the first two blood samples was sent out for metabolomic analysis.

### Metabolomic Analysis

#### Sample Processing

Samples were prepared by mixing 30 μL of serum with 200 μL of acetonitrile and vacuum-filtered into polypropylene well plates that were sealed until analysis (Captiva ND 0.2 μm filter and collection plates, Agilent Technologies, Santa Clara, CA; EPS well plate seals, BioChromato, Fujisawa, Japan). Quality control (QC) samples were prepared from a sample pool prepared by combining small aliquots of the study samples. Samples from the same participant were placed next to each other within the analytical sequence, while the order of the first and second blood sample of each participant was randomly altered. Different study centers were spread randomly across the sequence. After randomization, samples were analyzed as a single uninterrupted batch with liquid chromatography–mass spectrometry system consisting of a 1290 Binary LC system, a Jet Stream electrospray ionization (ESI) source, and a 6550 QTOF mass spectrometer (Agilent Technologies). The Autosampler tray was kept refrigerated at 4 °C, and 2 μL of the sample solution was injected into an ACQUITY UPLC HSS T3 column (2.1 mm × 100 mm, 1.8 μm; Waters, Milford, MA). The column temperature was 45 °C, and the mobile phase flow rate was 0.4 mL/min, consisting of ultrapure water and LC-MS-grade methanol, both containing 0.1% (v/v) of formic acid. The gradient profile was as follows: 0–6 min: 5% → 100% methanol, 6–10.5 min: 100% methanol, 10.5–13.5 min: 5% methanol. The mass spectrometer was operated in positive polarity using the following conditions: drying gas (nitrogen) temperature 175 °C and flow 12 L/min, sheath gas temperature 350 °C and flow 11 L/min, nebulizer pressure 45 psi, capillary voltage 3500 V, nozzle voltage 300 V, and fragmentor voltage 175 V. Data was acquired using extended dynamic range mode across a mass range of 50–1200, with an acquisition rate of 1.67 Hz. Continuous mass axis calibration was performed with two reference ions (*m*/*z* 121.050873 and *m*/*z* 922.009798). A QC sample was analyzed after every 12 study samples.

Preprocessing of the acquired data was performed using Qualitative Analysis B.06.00, DA Reprocessor, and Mass Profiler Professional 12.1 software (Agilent Technologies). Recursive feature finding was employed to find compounds as singly charged proton adducts [M + H]^+^ over a mass range of 50–1000 Da. The initial processing was performed using a “find by molecular feature (MFE)” algorithm set to small molecules. Threshold values for mass and chromatographic peak heights were 1500 and 10 000 counts, respectively, with a compound quality score threshold at 80. Isotope peak spacing tolerance was 0.0025 *m*/*z* + 7 ppm, with the isotope model set to common organic molecules. The resulting features were aligned using 0.075 min and 15 ppm + 2 mDa windows for retention time and mass, respectively. Features existing in at least 2% of all of the samples were used as targets for a recursive feature extraction using a “find by formula (FBF)” algorithm, with match tolerances of ±10 ppm and ±0.04 min. Ion species were limited to [M + H]^+^, with a threshold for chromatographic peak height at 2000 counts. The resulting features were aligned using the same settings as above.

This resulted in 11 217 features identifiable by their mass and retention time. After excluding the features present in every blank sample, unless 5-fold greater in intensity in the samples, and removing the compounds that were not detected in at least 40% of the samples (a threshold we have used in previous metabolomic-wide association studies^21^), 4294 features remained.

#### Annotation

The features were searched against a database of metabolites known to be detectable with the assay used in this study. This database was constructed by combining the elemental composition and retention time of the metabolites identified to MSI levels 1 or 2 in previous studies, where the same laboratory assay was used for the analysis of human plasma or serum (see Table S1 in the Supporting Information for more details). 42 additional lipid targets were included based on matching of the accurate mass and MS/MS spectra by using Agilent Lipid Annotator 1.0 software as described earlier.^22^ The database was created using Agilent MassHunter PCDL Manager B.08.00 software, and searching was performed with Agilent IDBrowser B.08.00 identification module of the Mass Profiler Professional 14.9.1 software. The software uses isotope patterns associated with the feature for the determination of charge state, allowing more specificity than searching for matching accurate mass alone. Matching tolerance was ±10 ppm and ±0.15 min for the mass and retention times, respectively. Only singly charged [M + H]^+^ ions were allowed with up to 10 matches per target, ranked by score consisting of the closeness of mass, retention time, and isotope spacing and abundance when detected. For metabolites known to be better detected as ions other than [M + H]^+^, additional adducts were allowed: [M + Na]^+^, [M-NH_3_ + H]^+^, [M]^+^, [M-H_2_O + H]^+^. These metabolites were 2-hydroxy-3-methylbutyric acid, α-tocopherol, docosahexaenoic acid, ethyl glucoside, γ-CEHC, glycoursodeoxycholic acid, inosine, serotonin, trigonelline, and valine.

In some cases, multiple features referred to the same compound (being either different ions, isomers fitting with the same annotation, or duplicate features due to the algorithm anomalies), in that case, we only reported the result of the feature with the highest ICC value.

#### Grouping Compounds by Chemical Class and Biological Pathway

Chemical classes were based on the ChEBI ontology.^23^ After retrieving the parents in the ontology from each compound, we looked for meaningful terms that were mutually exclusive and covered as many compounds as possible. Terms had to have at least seven members to be considered. Information on biological pathways a chemical compound was active in was retrieved from the KEGG database.^24^

### Statistical Methods

We used a linear mixed effects model with censored responses (multilevel tobit model) to estimate the variance components of the features. We defined a three-level nested random-intercept model that takes the nesting of subjects into centers into account:
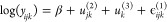
1for intensity measurements *i* = 1,···, *n*_*jk*_ and level-2 groups (subjects) *j* = 1,···,*M*_1*k*_ nested within level-3 groups (centers) *k* = 1,···, *M*_2_. Here, *u*_*jk*_^(2)^ is a level-2 random intercept, *u*_*k*_^(3)^ is a level-3 random intercept, and ϵ_*ijk*_^(1)^ is a level-1 error term (within-subject error). We assumed the error term and level-2 and level-3 random intercepts to be normally distributed with a mean of 0 and variances σ_1_^2^, and σ_2_^2^ and σ_3_^2^, respectively. All error terms and random intercepts were assumed to be independent of each other.

We assumed the feature intensity *y* to be left-censored at the limit of detection (LOD), which we defined as the lowest detected value for a compound. The models were implemented in R (version 4.2.1) using the brms package (version 2.17) which provides an interface to fit Bayesian models using the full Bayesian inference tool Stan.^25−27^ Coding scripts to reproduce the statistical analysis is available at https://doi.org/10.5281/zenodo.8247461.

From this model, we calculated the following intraclass correlation coefficient
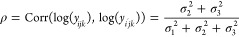
2This coefficient relates measurements from the same subject and center to measurements of different subjects and different centers. In our setting, this ICC corresponds to the correlation between measurements *i* and *i*’ from the same level-3 group (center) *k* and level-2 group (subject) *j.*(28) The calculation implicitly assumes that the between-center differences reflect true biological differences. We used the default weakly informative priors from brms and calculated the median ICCs from draws of the posterior of every compound. This ensured that the estimates were representative of the joint posterior if the posterior of the parameters were correlated. Both models ran for 10 000 iterations each with four chains, with the default number of burn-in samples (i.e., 5000 in this case). The adapt delta parameter was set to 0.99. All (reported) correlations/ICCs are on the natural logarithm scale.

An ICC below 0.40 was taken as poor repeatability, values between 0.40 and 0.75 as fair, and ICCs above 0.75 were taken to represent excellent repeatability.^29^

For the identified chemical compounds, we also estimated the ICC from a model that included fixed effects for a smooth term for age, body mass index (BMI), and sex, which represents the ICC that is relevant for a setting in which the epidemiological analysis is corrected for these potentially confounding factors. In addition to the ICC we report the proportion of variance attributable to between-subject, between-center, and within-subject variation for all metabolites.

#### Sensitivity Analyses

To assess the sensitivity of the estimated metabolite ICC to our decision to fit a three-level nested random-intercept model and to obtain model convergence for all features, we also fitted a two-level model to all features, in which we did not explicitly adjust for the multicenter design (further details in Supporting Methods 1). Using this model, we also investigated if repeatability was different on the transformed scale (natural logarithm) or the original, back-transformed scale (calculation method not published for three-level model). Further details can be found in Supporting Methods 1 and 2. Lastly, we calculated ICCs stratified by center for all identified compounds to investigate if repeatability differed by center (Supporting Methods 1).

#### Comparison to ICCs Reported Based on Targeted Assays

To compare the ICCs from our untargeted platform to targeted assays, we looked for targeted LC-MS studies that calculated ICCs of compounds in blood over a comparable time span (3–4 months), and a short time span (weeks), and found two comparable studies for a comparable time span^14,15^ and one study with a short time span.^11^ Floegel and colleagues analyzed (fasted) serum samples using BIOCRATES AbsoluteIDQ p150, while Yin et al. and Breier et al. analyzed (fasted) plasma samples with the BIOCRATES AbsoluteIDQ p180 kit. Subsequently, we matched the compounds they reported to our identified compounds.

## Results

### Study Population

Metabolomic data was available for 157 participants of the study. Of those, 141 subjects had two measurements and 16 one measurement. 48 of the subjects were recruited by the center in Basel, 25 in Norwich, 43 in Turin, and 41 in Utrecht. Baseline characteristics of the individuals are shown in Table 1. In brief, 61% of participants were female, and the average age was 60.5 (standard deviation (SD) 6.6). A majority had a university undergraduate degree or higher as their highest level of completed education. The median BMI was 25.3 (SD 4.1), and the average number of days between measurements was 107 (interquartile range (IQR) 34).

**Table 1:** Characteristics of the Subjects That Provided Blood Samples in the Personal Exposure Monitoring Study (PEM) From EXPOsOMICS^a^

	overall, *N* = 157^b^	Basel, *N* = 48^b^	Norwich, *N* = 25^b^	Turin, *N* = 43^b^	Utrecht, *N* = 41^b^
sex					
female	96 (61%)	23 (48%)	17 (68%)	22 (51%)	34 (83%)
age (years)	60.5 (6.6)	60.3 (8.5)	60.5 (5.1)	59.7 (4.6)	61.7 (6.5)
BMI (kg/m∧2)	25.3 (4.1)	24.8 (4.1)	26.7 (3.8)	25.2 (4.4)	25.1 (3.8)
highest level of education					
any secondary school	8 (5.1%)	1 (2.1%)	0 (0%)	6 (14%)	1 (2.4%)
high school	44 (28%)	3 (6.2%)	11 (44%)	24 (56%)	6 (15%)
university or higher	105 (67%)	44 (92%)	14 (56%)	13 (30%)	34 (83%)
samples available per participant					
1	16 (10%)	5 (10%)	8 (32%)	1 (2.3%)	2 (4.9%)
2	141 (90%)	43 (90%)	17 (68%)	42 (98%)	39 (95%)
date of first session	2014-03-25 [112]	2014-02-15 [51]	2014-06-02 [64]	2014-02-27 [38]	2014-06-26 [51]
date of second session	2014-07-08 [110]	2014-06-11 [48]	2014-09-08 [84]	2014-06-12 [42]	2014-10-09 [42]
days between measurements	107 [34]	113 [42]	92 [33]	105 [21]	103 [52]

a BMI = body mass index, kg = kilogram, m = meter.

b *n* (%); Mean (SD); Median [interquartile range in days].

### Assessment of the Blood Metabolome

From the 4294 features, 206 could be confidently identified (MSI requirement 1 and 2).^30^ These features referred to 184 unique compounds. Our grouping by class method identified five distinct chemical classes, covering 124 compounds in total. Glycerophospholipids were the most prevalent (*n* = 44), followed by phosphatidylcholines (*n* = 34), O-acylcarnitine (*n* = 30), amino acids (*n* = 9), and steroids (*n* = 7). Moreover, 18 exogenous compounds (compounds that the human body cannot produce) were identified (Table S2). These exogenous compounds consisted of essential amino acids, vitamins, and other dietary compounds. Some of these metabolites of dietary compounds are formed in the gut microbiota.

In total, 22 of the identified compounds had a KEGG entry with corresponding pathway entries. From these, only three KEGG pathways contained four compounds or more. Bile secretion was the most prevalent among the identified pathways with 7 compounds, followed by caffeine metabolism (5 compounds) and tryptophan metabolism (4 compounds). A full list of the confidently identified compounds, their mass and retention time, chemical class, and involved pathways can be found in Tables S1 and S3 of the Supporting Information.

69% of the nonidentified compounds and 50% of the identified features were not present in all samples (Figure S1).

### ICCs Per Compound, Class/Exposure Route, and Biological Pathway

**Figure 1:**
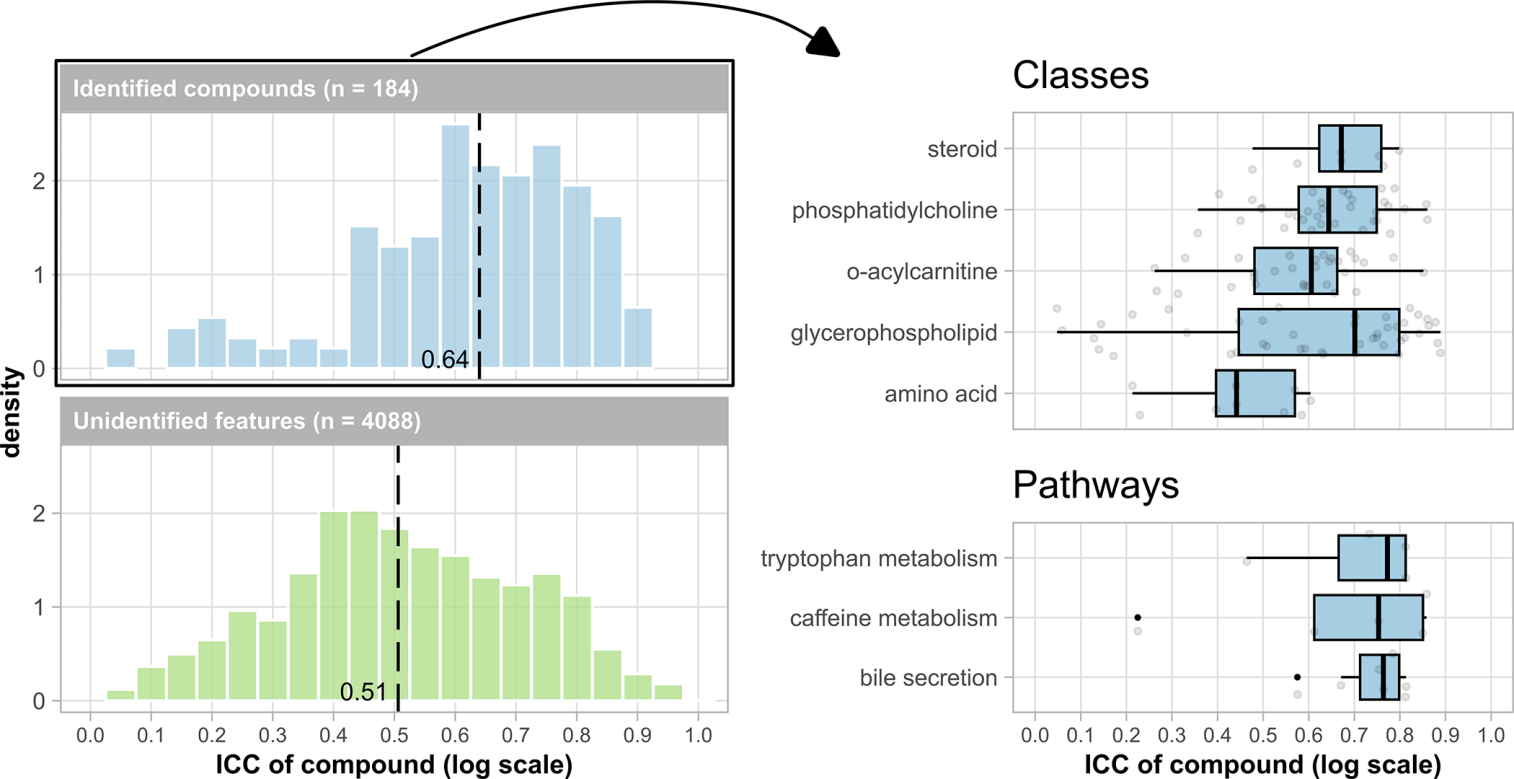
Distribution of ICC values from the unadjusted, three-level tobit model of the paper (eq 1). The dotted line in the histogram shows the median. The boxplots group the results from the identified compounds according to their chemical class, and the pathway they are involved in. Only pathways with at least four entries are shown. The transparent dots in the boxplot are jitter and show all individual data points. ICC = intraclass correlation coefficient.

Figure 1 (left) shows the distribution of the ICC values estimated using our model. The median ICC across all 4294 metabolic features detected using our HRMS approach was 0.51 (IQR 0.29). For the 184 identified chemical compounds, the median ICC was 0.64 (IQR 0.25).

α-Tocopherol, oleoylcarnitine, LysoPC (20:4), l,l-cyclo(Ile-Pro), LysoPC (20:3), LysoPC (18:1), 2-hydroxy-3-methylbutyric acid, LysoPC (16:0), trigonelline, and PC (36:1) were the 10 compounds with the highest estimated repeatability (ICC values ranging from 0.86 to 0.91). LysoPC (14:0), LysoPC (16:0), LysoPC (20:3), LysoPC (20:5), LysoPC (20:3), LysoPC (18:4), uric acid, trimethylamine N-oxide, methionine, and LysoPC (20:3) had the lowest estimated repeatability (ICC values ranging from 0.05 to 0.21).

In the boxplot of Figure 1, we stratified the results of the identified compounds according to their chemical class. In brief, there was a great variety in ICC values within all classes, with a difference of at least 0.3 between the highest and lowest ICC of the compounds in that class. The median ICC was highest for glycerophospholipid (0.70), followed by the steroid class (0.67) and the phosphatidylcholine class (0.64). The median ICC was lowest in the O-acylcarnitine class (0.61), and amino acid class (0.44). The average ICC for exogenous compounds was not remarkably different (0.61). All identified pathways had an average ICC of ca. 0.76.

Figures S2 and S3 show the relative size of the within-subject, between-subject, and between-center variance components for all features. In brief, the between-center variance was 6% of the total variance on average. The ICC was lower when we only compared subjects to subjects within the same center (bottom left plot in Figure S2, median ICC 0.42).

### Sensitivity of the Calculated ICCs to Adjustment for Common Confounders

The median ICC for the identified compounds after correcting for age, BMI, and sex in the regression model did not materially change the ICC (median 0.63 (IQR 0.24), vs 0.64 (IQR 0.25) for unadjusted). Detailed results of this model are presented in Figures S4 and S5. Including the original traffic condition (high vs low) besides the confounders did not change the ICC of the adjusted model notably (median 0.64 (IQR 0.23)).

### Sensitivity Analyses

ICCs for the compounds calculated using the two-level model were comparable to those calculated using our main model (median ICC: 0.60 on the natural logarithm scale and 0.57 on the data scale for the identified compounds; Figure S6). ICCs were similar across the four centers (Figure S7).

Model diagnostics were considered sufficient for all models (see the Model Diagnostics section in the Supporting Information).

### Comparison with Targeted Assays

For nine of the 184 compounds with confirmed identities from our study, we were able to retrieve ICCs for measurements in peripheral blood using the BIOCRATES kit from the literature.^14,15^ For six compounds, our ICCs were lower than reported in studies with comparable time frames (citrulline, methionine, proline, tryptophan, tyrosine, valine), and in three cases, our ICCs were higher (isoleucine, leucine, phenylalanine), but in general, the confidence intervals overlapped, and averages were in the same range (median ICC Floegel 0.54, Yin 0.52, our results 0.44). The repeatability was greatest in the study over the shortest time span (Breier 0.67). Detailed results can be found in Figure S8.

The ICCs and the 95% credibility interval (main and adjusted model) of the identified compounds are available in Table S4. The full results for each compound, all variance components, convergence statistics, and resulting ICC per compound are available in the online repository https://doi.org/10.5281/zenodo.8247461.

## Discussion

In this work, we assessed the variability of the features measured by untargeted LC-HRMS in serum samples repeatedly collected approximately 107 days apart. Our analyses indicated fair ICCs (median 0.64) for a set of 184 identified compounds. However, there was a considerable range in ICCs. This range remained after stratifying the results by chemical class and biological pathway. Differences between subjects’ metabolite levels were not explained by differences in age, sex, and BMI (average ICC: 0.63).

These findings are largely in line with other studies on the repeatability of LC-MS serum measurements. Sampson et al. also found a relatively small contribution of age and sex to variability in compound levels, while Townsend et al. reported similar average values for the amino acids and lipid classes (9, 10). Our findings, based on measures over a period of 3–4 months, are comparable to the repeatability reported by others covering periods of 1–2 years suggesting that our results can possibly be generalized to a longer time span.^9,13,31^ Compared to targeted assays over comparable time spans, we found lower repeatability values for amino acids. However, the differences were not large and expected when comparing targeted assays with untargeted assays. These results indicate that using LC-HRMS metabolomics may have a favorable trade-off between being broad (untargeted) while still reasonably repeatable on established markers. Compared to a targeted assay analysis over a period of 2 weeks,^11^ studies over 3–4 months find lower repeatability for the amino acids. This pattern would be reasonably explained by fewer environmental changes. If the factors that impact the measured metabolite levels are known (such as the time of year for vitamin D levels), incorporating them into the statistical model could reduce the within-subject variation and thereby improve the repeatability.

An important advantage of our study is that it is based on samples from a relatively large multicenter study that were collected under real-life circumstances. Therefore, our results are likely to be relevant for ongoing and planned exposome studies in which samples are collected following similar protocols. Additional strengths of this study include the explicit modeling of the multicentered setup, which allowed us to provide further insight into the observational and experimental contributions to the variability. Future studies can use these variability components and the ICCs to decide which compounds are stable (or volatile) enough to assess in a study.

There are several additional points about our study worth noting. First, because the features that can be annotated depend on the laboratory, the annotated set and its repeatability should not be viewed as an absolute, fixed set, but in the context of the laboratory, year of analyses, and the platform. Second, while we found only a very small impact of adjustment of age on average ICC values, it should be noted that the age range in this study was limited to individuals between 50 and 70 years old. Studying metabolomic samples from a more diverse age range may lead to a greater impact of age adjustment on the ICC. Lastly, in this multicenter study, characteristics like blood drawing, storage conditions, and time frame were harmonized, which may not be true for study efforts that combine archived samples from historical cohorts. In such a study, between-country differences may not reflect true biological differences, but instead differences in protocols. As a result, the ICC would be lower, but not considerably lower (as illustrated in Figure S2).

In conclusion, our results suggest that more than 50% of the metabolites measured using untargeted LC-HRMS, including the 184 chemicals that could be annotated, are sufficiently stable to reflect average levels over 100 days. The fair comparison in repeatability with targeted platform indicates that untargeted LC-HRMS might be a reasonable compromise between a broad scope while still sufficiently repeatable to quantify established risk factors.

## Data Availability

A dataset with variables to reproduce the main analysis is available at https://doi.org/10.5281/zenodo.8156759. Coding scripts to reproduce the main statistical analysis are available in a GitHub repository at https://doi.org/10.5281/zenodo.8247461.

## Supporting Information Available

Supporting methods, model diagnostics, additional tables and figures describing results in more detail (PDF)

## Notes

The authors declare that they have no known competing financial interests or personal relationships that could have appeared to influence the work reported in this paper. Where authors are identified as personnel of the International Agency for Research on Cancer/World Health Organization, the authors alone are responsible for the views expressed in this article, and they do not necessarily represent the decisions, policy, or views of the International Agency for Research on Cancer/World Health Organization.
