# Correction to “Variability of the Human Serum Metabolome over 3 Months in the EXPOsOMICS Personal Exposure Monitoring Study”

**DOI:** 10.1021/acs.est.5c14701

**Published:** 2026-03-03

**Authors:** Max J. Oosterwegel, Dorina Ibi, Lützen Portengen, Nicole Probst-Hensch, Sonia Tarallo, Alessio Naccarati, Medea Imboden, Ayoung Jeong, Nivonirina Robinot, Augustin Scalbert, Andre F. S. Amaral, Erik van Nunen, John Gulliver, Marc Chadeau-Hyam, Paolo Vineis, Roel Vermeulen, Pekka Keski-Rahkonen, Jelle Vlaanderen

In the abstract, we incorrectly described the type of measurement error observed across the metabolome as "differential measurement error". The correct term is "nondifferential measurement error". The final sentence of the abstract should read as follows: "Variance across the metabolome will result in nondifferential measurement error across the metabolome, which needs to be considered in the interpretation of metabolome results". This correction does not affect any data, results, or interpretations presented in the paper. We regret this error and appreciate the opportunity to correct the record.

