# Supplementary material for: Variability of the Human Serum Metabolome over 3 Months in the EXPOsOMICS Personal Exposure Monitoring Study
Source: Environ Sci Technol. 2023 Aug 15;57(34):12752–9. doi: 10.1021/acs.est.3c03233 (PMC10469440; doi:10.1021/acs.est.3c03233)
Supplement: Supplementary file 1 — es3c03233_si_001.pdf [file es3c03233_si_001.pdf]

Variability of the human serum metabolome over three months in the EXPOsOMICS Personal Exposure Monitoring study – Supplementary Materials

Max J Oosterwegel [a], Dorina Ibi [a], Lützen Portengen [a], Nicole Probst-Hensch [d,e], Sonia Tarallo [f], Alessio Naccarati [f], Medea Imboden [d,e], Ayoung Jeong [d,e], Nivonirina Robinot [i], Augustin Scalbert [i], Andre F S Amaral [h, k], Erik van Nunen [a], John Gulliver [c, j], Marc Chadeau-Hyam [a, c], Paolo Vineis [c,f], Roel Vermeulen [a,b,c], Pekka Keski-Rahkonen [i], Jelle Vlaanderen[a*]

[a] *Division of Environmental Epidemiology, Institute for Risk Assessment Sciences, Utrecht University, Utrecht, 3584 CM, The Netherlands.*

[b] *Julius Center for Health Sciences and Primary Care, University Medical Center Utrecht, Utrecht, 3508 GA, The Netherlands.*

[c] *Medical Research Council-Public Health England Center for Environment and Health, Department of Epidemiology and Biostatistics, Imperial College London, London, SW7 2AZ, United Kingdom.*

[d] *Swiss Tropical and Public Health Institute, Allschwil, 4123, Switzerland.*

[e] *University of Basel, Basel, 4001, Switzerland.*

[f] *Italian Institute for Genomic Medicine (IIGM), c/o IRCCS, Turin, 10060, Italy*

[h] *National Heart and Lung Institute, Imperial College London, London, SW3 6LY, UK.*

[i] *Nutrition and Metabolism Branch, International Agency for Research on Cancer, World Health Organization, Lyon, CS 90627, France.*

[j] *Centre for Environmental Health and Sustainability & School of Geography, Geology and the Environment, University of Leicester, Leicester, LE1 7RH, United Kingdom*

[k] *NIHR Imperial Biomedical Research Centre, London, W2 1NY, UK*

** Corresponding author. Email address: j.j.vlaanderen@uu.nl*

Summary: 28 pages, 4 tables, 8 figures

Supplementary Methods 1: Two-level model

We performed two additional sensitivity analyses using a two-level model tobit model with censored responses:

$$\log\left(y_{ij}\right) = \beta + u_j^{(2)} + \epsilon_{ij}^{(1)} \#$$

for intensity measurements $i = 1, \ldots n_j$ and level-2 groups (subjects) $j = 1, \ldots M$. Here β is an unknown fixed intercept, $u_j^{(2)}$ is a level-2 random intercept (between-subject error) and $\epsilon_{ij}^{(1)}$ is a level-1 error term (within-subject error). The random intercept and error terms are assumed to independent and normally distributed with mean 0 and variances σ_2^2 and σ_1^2 respectively.

Supplementary Methods 2: Logarithm scale versus data scale

To investigate whether repeatability was different on the natural logarithm scale compared to the data scale, we transformed the fitted parameters from the two-level model (see Supplementary Methods 1) to the data scale as follows. First, to obtain the expected mean on the data scale we averaged the $\exp\left(\mu + \frac{\sigma_1^2}{2}\right)$ transformation over the random effects distribution

$$\mathrm{E}(y) = \bar{\mu} = \int \exp\left(x + \frac{\sigma_1^2}{2}\right) f_{\mathcal{N}}\left(x, \beta, \sigma_2^2\right) dx$$

Using this marginal mean, we can calculate the expected variance between subjects on the data scale

$$\sigma_B^2 = \int (\exp(x) - \bar{\mu})^2 f_{\mathcal{N}}\left(x, \beta, \sigma_2^2\right) dx$$

Lastly, we obtain the expected variance within subjects on the data scale by

$$\sigma_W^2 = \int \left(\left(\exp\left(\sigma_1^2\right) - 1\right) \exp\left(2x + \sigma_1^2\right)\right) f_{\mathcal{N}}\left(x, \beta, \sigma_2^2\right) dx$$

ICCs on the data scale could then simply calculated as between-subject variance as proportion of total variance:

$$\mathrm{Cor}\left(y_{ij}, y_{ij'}\right) = \frac{\sigma_B^2}{\sigma_B^2 + \sigma_W^2}$$

[1,2]

Model Diagnostics

Average model diagnostics were deemed sufficient. In the unadjusted, three-level tobit model of the paper (equation 1 paper), $\hat{R}$ values were below 1.05 for all calculated ICCs except for six of them, indicating that the chains converged (approximately) in 99.86% of the fitted models. Bulk-ESS and tail-ESS for the calculated ICCs were at least hundred times the number of chains (400) for 99.13% and 98.10% of the compounds respectively indicating that the variance and median estimates of the posterior were reliable for the vast majority of the compounds. For the model where we have added fixed effects for age, BMI and sex to the three-level tobit model of the paper (equation 1), the bulk-ESS and $\hat{R}$ statistics were sufficient for all compounds, while tail-ESS was sufficient for 99.46% of compounds. All fits of the two-level tobit-model (see Supplementary Methods 1) had sufficient model diagnostics.

Table S1: Monoisotopic mass, retention time and classes of compounds that could be identified. The identity column describes if two annotated features with the same name, refer to the compound (a, a) or different (a, b). For example, we have three annotations of ‘Oleoylcarnitine’ at three different retention times (3.9859, 3.9825 and 4.1967). The first two annotations belong to the same compound (a, a) but the last annotation belongs to a different one (b). The MSI column refers to the MSI level of confidence of the annotation [3]. The ‘More info’ column describes where you can find more information on the annotation. If it only mentions a number it refers to the PubMed Identifier of the relevant publication. NA = not available.

Monoisotopic mass	Retention time	MSI	Annotation	Identity	ChEBI ID	More info	Class
202.0144	2.730	1	2-Hydroxy-3-methylbutyric acid		CHEBI:60645	34010397	
198.0750	0.856	1	5-Acetylamino-6-amino-3-methyluracil		CHEBI:80473	31483556	
329.2560	5.380	2	ACar 11:0		CHEBI:165621	35779425	O-acylcarnitine
327.2402	5.190	2	ACar 11:1		CHEBI:190572	35779425	O-acylcarnitine
357.2868	5.830	2	ACar 13:0		CHEBI:190643	35779425	O-acylcarnitine
355.2712	5.680	2	ACar 13:1		NA	35779425	
203.1159	0.719	2	Acetylcarnitine		CHEBI:73024	28122782	O-acylcarnitine
429.3722	8.840	1	alpha-Tocopherol	a	CHEBI:22470	Unpublished	
430.3797	8.840	1	alpha-Tocopherol	a	CHEBI:22470	Unpublished	
280.1054	2.660	2	Asp-Phe		CHEBI:73830	Unpublished	
584.2634	4.330	2	Bilirubin isomer 1		CHEBI:142137	32734650	
584.2635	5.130	2	Bilirubin isomer 2		CHEBI:16990	32734650	
231.1465	1.950	2	Butyrylcarnitine	a	CHEBI:7676	29401400	O-acylcarnitine
231.1467	2.000	2	Butyrylcarnitine	b	CHEBI:7676	35779425	O-acylcarnitine
194.0807	3.180	1	Caffeine		CHEBI:27732	33669644	
368.3439	9.060	1	Cholesterol		CHEBI:16113	32553738	steroid
175.0962	0.629	1	Citrulline		CHEBI:18211	Unpublished	amino acid
362.2089	5.170	1	Cortisol		CHEBI:17650	29179034	steroid
360.1925	5.010	1	Cortisone		CHEBI:16962	29179034	steroid
131.0693	0.661	1	Creatine		CHEBI:16919	32734650	
244.1217	3.940	1	Cyclo(Phe-Pro)	a	CHEBI:69440	31168595	
244.1204	4.050	1	Cyclo(Phe-Pro)	b	CHEBI:69440	31168595	
614.4867	9.770	2	DAG(34:2)	a	CHEBI:85693	29401400	
614.4874	9.770	2	DAG(34:2)	a	CHEBI:85693	29401400	
638.4873	9.410	2	DAG(36:4)		CHEBI:85699	29401400	
311.2097	4.580	2	Decadienylcarnitine	a	CHEBI:190630	35779425	O-acylcarnitine
311.2089	4.630	2	Decadienylcarnitine	b	CHEBI:190630	35779425	O-acylcarnitine

315.2407	5.190	1	Decanoylcarnitine		CHEBI:68830	29401400	O-acylcarnitine
313.2245	4.920	2	Decenoylcarnitine	a	CHEBI:86063	29401400	O-acylcarnitine
313.2250	4.920	2	Decenoylcarnitine	a	CHEBI:86063	29401400	
313.2247	5.000	2	Decenoylcarnitine	b	CHEBI:86063	29401400	O-acylcarnitine
94.0088	0.835	2	Dimethylsulfone		CHEBI:9349	Agilent 5994-0775EN	
350.2224	7.230	1	Docosahexaenoic acid	a	CHEBI:36005	29401400	
328.2399	7.230	1	Docosahexaenoic acid	a	CHEBI:36005	29401400	
339.2389	5.290	2	Dodecadienylcarnitine		NA	35779425	
343.2717	5.690	1	Dodecanoylcarnitine		CHEBI:73054	35779425	O-acylcarnitine
341.2555	5.470	2	Dodecenoylcarnitine	a	CHEBI:190666	29401400	O-acylcarnitine
341.2567	5.540	2	Dodecenoylcarnitine	b	CHEBI:190666	29401400	O-acylcarnitine
341.2558	5.540	2	Dodecenoylcarnitine	b	CHEBI:190666	29401400	
230.0760	1.400	1	Ethyl glucoside	a	NA	34010397	
230.0762	0.859	1	Ethyl glucoside	a	NA	34010397	
263.1285	5.260	1	gamma-CEHC		CHEBI:89379	32734650	
449.3132	6.420	1	Glycochenodeoxycholic acid		CHEBI:36274	32734650	steroid
465.3092	6.150	1	Glycocholic acid		CHEBI:17687	31168595	steroid
449.3142	6.500	1	Glycodeoxycholic acid		CHEBI:27471	32734650	steroid
431.3000	5.870	1	Glycoursodeoxycholic acid		CHEBI:89929	Unpublished	steroid
283.0910	1.650	1	Guanosine		CHEBI:16750	28391173	
399.3341	6.300	2	Hexadecanoylcarnitine		CHEBI:73067	35779425	O-acylcarnitine
393.2872	5.880	2	Hexadecatrienylcarnitine		NA	Agilent 5994-0775EN	
397.3177	6.140	2	Hexadecenoylcarnitine		CHEBI:86032	29401400	O-acylcarnitine
259.1776	3.360	2	Hexanoylcarnitine		CHEBI:70749	33669644	O-acylcarnitine
179.0582	3.070	1	Hippuric acid		CHEBI:18089	33669644	
385.2830	5.610	2	Hydroxytetradecenoylcarnitine		CHEBI:86067	29401400	O-acylcarnitine
136.0381	0.858	1	Hypoxanthine	a	CHEBI:17368	30665271	
136.0382	1.140	1	Hypoxanthine	a	CHEBI:17368	30665271	
175.0640	4.120	1	Indole-3-acetic acid		CHEBI:16411	33669644	
189.0797	4.560	1	Indole-3-propionic acid		CHEBI:43580	33669644	
205.0744	3.830	1	Indolelactic acid		CHEBI:17282	29401400	
268.0809	1.640	1	Inosine	a	CHEBI:17596	Unpublished	
136.0381	1.640	1	Inosine	a	CHEBI:17596	Unpublished	
268.0804	0.861	1	Inosine	a	CHEBI:17596	Unpublished	
147.0322	3.310	1	Isatin		CHEBI:27539	32734650	
131.0947	1.380	1	Isoleucine		CHEBI:24898	36581893	amino acid

189.0416	3.060	1	Kynurenic acid		CHEBI:18344	30522001	
208.0843	1.850	1	Kynurenine		CHEBI:28683	29179034	
210.1374	3.860	1	L,L-Cyclo(Ile-Pro)		CHEBI:182600	32734650	
210.1374	3.760	1	L,L-Cyclo(Ile-Pro)		CHEBI:182600	Unpublished	
230.1622	2.280	2	Leu-Val		CHEBI:73579	32734650	
131.0946	1.470	1	Leucine		CHEBI:25017	32553738	amino acid
423.3339	6.230	2	Linoleylcarnitine		CHEBI:73072	35779425	O-acylcarnitine
467.3008	6.640	2	LysoPC (14:0)	a	CHEBI:64483	Agilent 5994-0775EN	glycerophospholipid
467.3007	6.720	2	LysoPC (14:0)	b	CHEBI:64483	32734650	glycerophospholipid
465.3161	6.720	2	LysoPC (14:0)	b	CHEBI:64483	32734650	
481.3154	6.870	2	LysoPC (15:0)		CHEBI:72736	32734650	glycerophospholipid
479.3004	7.050	2	LysoPC (15:1)		CHEBI:166929	Agilent 5994-0775EN	glycerophospholipid
495.3313	6.990	2	LysoPC (16:0)		CHEBI:64563	32734650	glycerophospholipid
495.3317	6.930	2	LysoPC (16:0)		CHEBI:64563	32734650	glycerophospholipid
523.2947	6.990	2	LysoPC (16:0)		CHEBI:64563	32734650	glycerophospholipid
493.3163	6.810	2	LysoPC (16:1)		CHEBI:64560	29401400	glycerophospholipid
493.3171	6.740	2	LysoPC (16:1)		CHEBI:64560	29401400	glycerophospholipid
509.3470	7.110	2	LysoPC (17:0)	a	CHEBI:72737	32734650	glycerophospholipid
507.3542	7.110	2	LysoPC (17:0)	a	CHEBI:72737	32734650	
507.3299	6.940	2	LysoPC (17:1)		CHEBI:73853	32734650	glycerophospholipid
523.3628	7.230	2	LysoPC (18:0)		CHEBI:64561	32734650	glycerophospholipid
521.3473	6.980	2	LysoPC (18:1)		CHEBI:64566	29401400	glycerophospholipid
521.3470	7.050	2	LysoPC (18:1)		CHEBI:64566	32734650	glycerophospholipid
519.3317	6.900	2	LysoPC (18:2)	a	CHEBI:64549	32734650	glycerophospholipid
519.3316	6.840	2	LysoPC (18:2)	b	CHEBI:64549	32734650	glycerophospholipid
517.3139	6.930	2	LysoPC (18:3)	a	CHEBI:64565	29401400	glycerophospholipid
517.3140	6.990	2	LysoPC (18:3)	b	CHEBI:64565	29401400	glycerophospholipid
515.2982	6.820	2	LysoPC (18:4)	a	CHEBI:132547	Agilent 5994-0775EN	glycerophospholipid
515.3014	6.730	2	LysoPC (18:4)	b	CHEBI:132547	Agilent 5994-0775EN	glycerophospholipid
547.3612	7.130	2	LysoPC (20:2)		CHEBI:67056	29401400	glycerophospholipid
543.3661	7.230	2	LysoPC (20:3)		CHEBI:64481	Agilent 5994-0775EN	glycerophospholipid
545.3476	7.000	2	LysoPC (20:3)	a	CHEBI:64481	32734650	glycerophospholipid
545.3517	6.920	2	LysoPC (20:3)	b	CHEBI:64481	32734650	glycerophospholipid
545.3452	7.160	2	LysoPC (20:3)	c	CHEBI:64481	32734650	glycerophospholipid

545.3456	7.230	2	LysoPC (20:3)	d	CHEBI:64481	32734650	glycerophospholipid
545.3443	7.680	2	LysoPC (20:3)	e	CHEBI:64481	32734650	glycerophospholipid
543.3316	6.890	2	LysoPC (20:4)	a	CHEBI:64568	32734650	glycerophospholipid
543.3296	7.060	2	LysoPC (20:4)	b	CHEBI:64568	32734650	glycerophospholipid
541.3161	6.740	2	LysoPC (20:5)	a	CHEBI:64559	32734650	glycerophospholipid
541.3139	6.900	2	LysoPC (20:5)	b	CHEBI:64559	32734650	glycerophospholipid
541.3143	6.820	2	LysoPC (20:6)		NA	32734650	
569.3476	6.960	2	LysoPC (22:5)	a	CHEBI:74349	32734650	glycerophospholipid
569.3490	6.950	2	LysoPC (22:5)	a	CHEBI:74349	32734650	
569.3473	6.970	2	LysoPC (22:5)	b	CHEBI:74349	32734650	glycerophospholipid
567.3338	6.880	2	LysoPC (22:6)		CHEBI:64567	32734650	glycerophospholipid
453.2849	6.930	2	LysoPE (16:0)	a	CHEBI:64563	Agilent 5994-0775EN	glycerophospholipid
453.2847	6.990	2	LysoPE (16:0)	b	CHEBI:64563	Agilent 5994-0775EN	glycerophospholipid
451.2692	6.810	2	LysoPE (16:1)	a	CHEBI:145247	Unpublished	glycerophospholipid
451.2709	6.870	2	LysoPE (16:1)	b	CHEBI:145247	Unpublished	glycerophospholipid
477.2848	6.900	2	LysoPE (18:2)	a	CHEBI:91296	Agilent 5994-0775EN	glycerophospholipid
477.2848	6.840	2	LysoPE (18:2)	b	CHEBI:91296	Agilent 5994-0775EN	glycerophospholipid
501.2849	6.890	2	LysoPE (20:4)		CHEBI:64569	30665271	glycerophospholipid
525.2870	6.880	2	LysoPE (22:6)		CHEBI:72734	32734650	glycerophospholipid
149.0504	0.850	1	Methionine		CHEBI:16811	Unpublished	amino acid
187.1683	0.518	1	N1-Acetylspermidine		CHEBI:17927	32734650	
301.2245	4.670	2	Nonanoylcarnitine	a	CHEBI:85527	29179034	O-acylcarnitine
301.2252	4.750	2	Nonanoylcarnitine	b	CHEBI:85527	29179034	O-acylcarnitine
301.2245	4.860	2	Nonanoylcarnitine	c	CHEBI:85527	29179034	O-acylcarnitine
425.3496	6.360	2	O-Palmitoleoylcarnitine		CHEBI:73066	35779425	O-acylcarnitine
287.2091	4.460	2	Octanoylcarnitine		CHEBI:18102	29401400	O-acylcarnitine
285.1932	3.990	2	Oleoylcarnitine	a	CHEBI:72689	Unpublished	O-acylcarnitine
285.1940	3.980	2	Oleoylcarnitine	a	CHEBI:72689	Unpublished	
285.1935	4.200	2	Oleoylcarnitine	b	CHEBI:72689	Unpublished	O-acylcarnitine
180.0655	2.680	1	Paraxanthine		CHEBI:25858	31483556	
705.5317	8.460	2	PC (30:0)		CHEBI:65303	32553738	phosphatidylcholine
703.5154	8.180	2	PC (30:1)		CHEBI:65302	Agilent 5994-0775EN	phosphatidylcholine
733.5628	8.940	2	PC (32:0)		CHEBI:66850	29401400	phosphatidylcholine
731.5457	8.550	2	PC (32:1)		CHEBI:66849	32734650	phosphatidylcholine

729.5325	8.280	2	PC (32:2)		CHEBI:66848	32734650	phosphatidylcholine
745.5618	8.770	2	PC (33:1)		CHEBI:86472	Agilent 5994-0775EN	phosphatidylcholine
743.5464	8.460	2	PC (33:2)		CHEBI:85853	Agilent 5994-0775EN	phosphatidylcholine
759.5760	9.030	2	PC (34:1)		CHEBI:64517	Agilent 5994-0775EN	phosphatidylcholine
757.5623	8.660	2	PC (34:2)	a	CHEBI:64516	29401400	phosphatidylcholine
389.7658	8.660	2	PC (34:2)	a	CHEBI:64516	29401400	
755.5469	8.400	2	PC (34:3)	a	CHEBI:64424	Agilent 5994-0775EN	phosphatidylcholine
755.5472	8.420	2	PC (34:3)	a	CHEBI:64424	Agilent 5994-0775EN	
753.5323	8.240	2	PC (34:4)		CHEBI:64423	Agilent 5994-0775EN	phosphatidylcholine
771.5769	8.940	2	PC (35:2)		CHEBI:85766	Agilent 5994-0775EN	phosphatidylcholine
787.6086	9.720	2	PC (36:1)	a	CHEBI:66857	Unpublished	phosphatidylcholine
787.6097	9.760	2	PC (36:1)	a	CHEBI:66857	Unpublished	
785.5942	9.220	2	PC (36:2)		CHEBI:64433	Agilent 5994-0775EN	phosphatidylcholine
783.5763	8.810	2	PC (36:3)		CHEBI:64523	Agilent 5994-0775EN	phosphatidylcholine
792.5513	8.610	2	PC (36:4)	a	CHEBI:64520	29401400	phosphatidylcholine
781.5631	9.050	2	PC (36:4)	b	CHEBI:64520	29401400	phosphatidylcholine
781.5615	8.470	2	PC (36:4)	a	CHEBI:64520	Agilent 5994-0775EN	phosphatidylcholine
781.5627	8.610	2	PC (36:4)	b	CHEBI:64520	Agilent 5994-0775EN	phosphatidylcholine
779.5494	8.390	2	PC (36:5)		CHEBI:64504	Agilent 5994-0775EN	phosphatidylcholine
777.5330	8.180	2	PC (36:6)		CHEBI:66856	Agilent 5994-0775EN	phosphatidylcholine
795.5762	8.790	2	PC (37:4)	a	CHEBI:72427	Agilent 5994-0775EN	phosphatidylcholine
795.5774	8.870	2	PC (37:4)	b	CHEBI:72427	Agilent 5994-0775EN	phosphatidylcholine
811.6084	9.470	2	PC (38:3)		CHEBI:64446	Unpublished	phosphatidylcholine
809.5939	9.160	2	PC (38:4)		CHEBI:64526	29401400	phosphatidylcholine
809.5909	8.950	2	PC (38:4)		CHEBI:64526	Agilent 5994-0775EN	phosphatidylcholine
807.5758	8.720	2	PC (38:5)	a	CHEBI:64525	Agilent 5994-0775EN	phosphatidylcholine
807.5799	8.830	2	PC (38:5)	b	CHEBI:64525	Agilent 5994-0775EN	phosphatidylcholine
805.5636	8.430	2	PC (38:6)	a	CHEBI:64519	32734650	phosphatidylcholine

805.5629	8.550	2	PC (38:6)	b	CHEBI:64519	32734650	phosphatidylcholine
805.5614	8.830	2	PC (38:6)		CHEBI:64519	Agilent 5994-0775EN	phosphatidylcholine
803.5453	8.240	2	PC (38:7)		CHEBI:64498	Agilent 5994-0775EN	phosphatidylcholine
835.6072	9.260	2	PC (40:5)		CHEBI:64524	Unpublished	phosphatidylcholine
833.5956	9.060	2	PC (40:6)	a	CHEBI:64431	Agilent 5994-0775EN	phosphatidylcholine
833.5906	9.460	2	PC (40:6)	b	CHEBI:64431	Agilent 5994-0775EN	phosphatidylcholine
831.5739	8.630	2	PC (40:7)		CHEBI:64521	Agilent 5994-0775EN	phosphatidylcholine
739.5203	8.610	2	PE (36:4)		CHEBI:134271	Agilent 5994-0775EN	glycerophospholipid
119.0738	3.660	2	Phe-Phe		CHEBI:72723	Unpublished	
264.1118	3.100	1	Phenylacetylglutamine		CHEBI:25982	33669644	
165.0791	2.060	1	Phenylalanine	a	CHEBI:28044	Unpublished	amino acid
165.0789	1.600	1	Phenylalanine	a	CHEBI:28044	Unpublished	amino acid
129.0792	0.844	1	Pipecolic acid	a	CHEBI:17964	Unpublished	
129.0788	0.845	1	Pipecolic acid	a	CHEBI:17964	Unpublished	
793.5989	9.070	2	Plasmalogen PC (38:4) or PC (O-38:5)		CHEBI:64445	32553738	glycerophospholipid
115.0634	0.685	1	Proline		CHEBI:26271	33669644	amino acid
217.1312	1.320	1	Propionylcarnitine	a	CHEBI:28867	29179034	O-acylcarnitine
217.1312	0.861	1	Propionylcarnitine	a	CHEBI:28867	29179034	
268.2196	7.220	1	Retinol		CHEBI:50211	32734650	
159.0688	1.510	1	Serotonin	b	CHEBI:28790	31168595	
159.0689	0.864	1	Serotonin	a	CHEBI:28790	31168595	
674.5346	8.100	2	SM d32:1		CHEBI:64586	Agilent 5994-0775EN	
672.5191	7.840	2	SM d32:2		CHEBI:72510	Agilent 5994-0775EN	
688.5500	8.260	2	SM d33:1		CHEBI:64585	Agilent 5994-0775EN	
702.5683	8.440	2	SM d34:1		CHEBI:72514	Agilent 5994-0775EN	
700.5514	8.170	2	SM d34:2		CHEBI:64587	Agilent 5994-0775EN	
728.5814	8.540	2	SM d36:2		CHEBI:72519	Agilent 5994-0775EN	
299.2818	6.110	1	Sphingosine		CHEBI:16393	32734650	
367.2708	5.670	2	Tetradecadienylcarnitine	a	CHEBI:192093	29401400	O-acylcarnitine
367.2712	5.680	2	Tetradecadienylcarnitine	a	CHEBI:192093	29401400	

367.2705	5.810	2	Tetradecadienylcarnitine	b	CHEBI:192093	29401400	O-acylcarnitine
371.3024	6.040	1	Tetradecanoylcarnitine		CHEBI:73061	29401400	O-acylcarnitine
369.2870	5.890	2	Tetradecenoylcarnitine	a	CHEBI:86066	29401400	O-acylcarnitine
369.2870	5.950	2	Tetradecenoylcarnitine	b	CHEBI:86066	29401400	O-acylcarnitine
180.0647	2.360	1	Theobromine		CHEBI:28946	33669644	
180.0651	2.800	1	Theophylline		CHEBI:28177	33669644	
137.0477	0.669	1	Trigonelline	a	CHEBI:18123	31168595	
159.0304	0.674	1	Trigonelline	a	CHEBI:18123	31168595	
75.0687	0.608	1	trimethylamine N-oxide		CHEBI:15724	28122782	
351.1580	3.610	2	Trp-Phe		CHEBI:74874	32734650	
204.0903	2.500	1	Tryptophan		CHEBI:27897	29179034	amino acid
181.0736	1.240	1	Tyrosine		CHEBI:18186	31168595	amino acid
168.0285	1.060	1	Uric acid		CHEBI:27226	29179034	
245.1628	2.620	2	Valerylcarnitine	a	CHEBI:86050	32734650	O-acylcarnitine
245.1627	2.570	2	Valerylcarnitine	b	CHEBI:86050	32734650	O-acylcarnitine
139.0614	0.806	1	Valine	a	CHEBI:27266	34253844	
117.0789	0.809	1	Valine	a	CHEBI:27266	30665271	amino acid

Table S2: all identified exogenous compounds (i.e. compounds that cannot be produced by the human body) and their respective class.

Annotation	Class
Valine	amino acid
Phenylalanine	amino acid
Tryptophan	amino acid
Leucine	amino acid
Isoleucine	amino acid
Methionine	amino acid
Indolelactic acid	
Indole-3-propionic acid	
Pipecolic acid	
alpha-Tocopherol	
Trigonelline	
5-Acetylamino-6-amino-3-methyluracil	
Retinol	
Indole-3-acetic acid	
Caffeine	
Theobromine	
Theophylline	
Paraxanthine	

Table S3: Annotation and corresponding KEGG pathway.

Annotation	KEGG entry	KEGG pathway name	KEGG pathway code
Hypoxanthine	C00262	Purine metabolism	map00230
Hypoxanthine	C00262	Nucleotide metabolism	map01232
Hypoxanthine	C00262	Purine metabolism	map00230
Hypoxanthine	C00262	Nucleotide metabolism	map01232
Serotonin	C00780	Tryptophan metabolism	map00380
Serotonin	C00780	cAMP signaling pathway	map04024
Serotonin	C00780	Neuroactive ligand-receptor interaction	map04080
Serotonin	C00780	Gap junction	map04540
Serotonin	C00780	Synaptic vesicle cycle	map04721
Serotonin	C00780	Serotonergic synapse	map04726
Serotonin	C00780	Taste transduction	map04742
Serotonin	C00780	Inflammatory mediator regulation of TRP channels	map04750
Serotonin	C00780	Bile secretion	map04976
Serotonin	C00780	Chemical carcinogenesis - receptor activation	map05207
Serotonin	C00780	Tryptophan metabolism	map00380
Serotonin	C00780	cAMP signaling pathway	map04024
Serotonin	C00780	Neuroactive ligand-receptor interaction	map04080
Serotonin	C00780	Gap junction	map04540
Serotonin	C00780	Synaptic vesicle cycle	map04721
Serotonin	C00780	Serotonergic synapse	map04726
Serotonin	C00780	Taste transduction	map04742
Serotonin	C00780	Inflammatory mediator regulation of TRP channels	map04750
Serotonin	C00780	Bile secretion	map04976
Serotonin	C00780	Chemical carcinogenesis - receptor activation	map05207
Inosine	C00294	Purine metabolism	map00230
Inosine	C00294	Nucleotide metabolism	map01232
Inosine	C00294	ABC transporters	map02010
Inosine	C00294	Purine metabolism	map00230
Inosine	C00294	Nucleotide metabolism	map01232
Inosine	C00294	ABC transporters	map02010

Annotation	KEGG entry	KEGG pathway name	KEGG pathway code
Inosine	C00294	Purine metabolism	map00230
Inosine	C00294	Nucleotide metabolism	map01232
Inosine	C00294	ABC transporters	map02010
Guanosine	C00387	Purine metabolism	map00230
Guanosine	C00387	Nucleotide metabolism	map01232
Guanosine	C00387	ABC transporters	map02010
Cortisol	C00735	Steroid hormone biosynthesis	map00140
Cortisol	C00735	Neuroactive ligand-receptor interaction	map04080
Cortisol	C00735	Cortisol synthesis and secretion	map04927
Cortisol	C00735	Cushing syndrome	map04934
Cortisol	C00735	Aldosterone-regulated sodium reabsorption	map04960
Cortisol	C00735	Bile secretion	map04976
Cortisol	C00735	Pathways in cancer	map05200
Cortisol	C00735	Prostate cancer	map05215
Cortisone	C00762	Steroid hormone biosynthesis	map00140
Cortisone	C00762	Aldosterone-regulated sodium reabsorption	map04960
Cortisone	C00762	Pathways in cancer	map05200
Cortisone	C00762	Prostate cancer	map05215
Kynurenic acid	C01717	Tryptophan metabolism	map00380
Glycocholic acid	C01921	Primary bile acid biosynthesis	map00120
Glycocholic acid	C01921	Bile secretion	map04976
Glycocholic acid	C01921	Cholesterol metabolism	map04979
5-Acetylamino-6-amino-3-methyluracil	C16366	Caffeine metabolism	map00232
Cholesterol	C00187	Steroid biosynthesis	map00100
Cholesterol	C00187	Primary bile acid biosynthesis	map00120
Cholesterol	C00187	Steroid hormone biosynthesis	map00140
Cholesterol	C00187	Ovarian steroidogenesis	map04913
Cholesterol	C00187	Aldosterone synthesis and secretion	map04925

Annotation	KEGG entry	KEGG pathway name	KEGG pathway code
Cholesterol	C00187	Cortisol synthesis and secretion	map04927
Cholesterol	C00187	Cushing syndrome	map04934
Cholesterol	C00187	Fat digestion and absorption	map04975
Cholesterol	C00187	Bile secretion	map04976
Cholesterol	C00187	Vitamin digestion and absorption	map04977
Cholesterol	C00187	Cholesterol metabolism	map04979
Cholesterol	C00187	Pathways in cancer	map05200
Cholesterol	C00187	Basal cell carcinoma	map05217
Cholesterol	C00187	Lipid and atherosclerosis	map05417
Glycochenodeoxycholic acid	C05466	Primary bile acid biosynthesis	map00120
Glycochenodeoxycholic acid	C05466	Bile secretion	map04976
Glycochenodeoxycholic acid	C05466	Cholesterol metabolism	map04979
Creatine	C00300	Arginine and proline metabolism	map00330
Sphingosine	C00319	Sphingolipid metabolism	map00600
Sphingosine	C00319	Sphingolipid signaling pathway	map04071
Sphingosine	C00319	Apoptosis	map04210
Sphingosine	C00319	Necroptosis	map04217
Indole-3-acetic acid	C00954	Tryptophan metabolism	map00380
Caffeine	C07481	Caffeine metabolism	map00232
Theobromine	C07480	Caffeine metabolism	map00232
Theophylline	C07130	Caffeine metabolism	map00232
Paraxanthine	C13747	Caffeine metabolism	map00232
Bilirubin isomer 2	C00486	Porphyrin metabolism	map00860
Bilirubin isomer 2	C00486	Bile secretion	map04976
Dimethylsulfone	C11142	Sulfur metabolism	map00920

Table S4: intraclass correlation coefficients (ICCs) of the identified compounds according to the ICC calculation from the unadjusted, three-level tobit model of the paper (equation 1 paper), and ICC according to the adjusted model of the paper where we have added fixed effects for age, BMI and sex to the three-level tobit model of the paper. Age was a smooth term with brms defaults. See online repository for full results. CI = credible interval.

Annotation	ICC	ICC adjusted model	95% CI of ICC	95% CI of ICC adjusted model
Bilirubin isomer 2	0.780	0.780	0.690 – 0.940	0.680 – 0.930
Indolelactic acid	0.820	0.810	0.740 – 0.950	0.730 – 0.950
PC (32:0)	0.360	0.340	0.190 – 0.550	0.170 – 0.550
Indole-3-propionic acid	0.650	0.630	0.540 – 0.820	0.490 – 0.860
Hippuric acid	0.610	0.580	0.480 – 0.770	0.440 – 0.780
PC (38:7)	0.760	0.770	0.640 – 0.920	0.650 – 0.920
PC (40:7)	0.630	0.620	0.500 – 0.870	0.480 – 0.890
PC (30:0)	0.610	0.610	0.460 – 0.880	0.460 – 0.900
Hydroxytetradecenoylcarnitine	0.450	0.460	0.300 – 0.660	0.300 – 0.770
PC (33:1)	0.680	0.690	0.510 – 0.940	0.520 – 0.960
Cyclo(Phe-Pro)	0.830	0.830	0.740 – 0.950	0.740 – 0.940
Cyclo(Phe-Pro)	0.700	0.680	0.570 – 0.860	0.540 – 0.880
PC (33:2)	0.750	0.750	0.630 – 0.950	0.620 – 0.950
PC (35:2)	0.590	0.550	0.450 – 0.870	0.400 – 0.850
PC (32:2)	0.560	0.560	0.400 – 0.870	0.400 – 0.890
PC (30:1)	0.660	0.660	0.520 – 0.880	0.510 – 0.900
PC (36:5)	0.810	0.800	0.680 – 0.970	0.670 – 0.970
PC (40:5)	0.570	0.570	0.370 – 0.930	0.360 – 0.920
PC (38:4)	0.790	0.790	0.710 – 0.900	0.710 – 0.900
LysoPC (16:0)	0.059	0.067	0.002 – 0.320	0.002 – 0.390
LysoPC (16:0)	0.860	0.870	0.810 – 0.940	0.810 – 0.950
LysoPC (16:0)	0.740	0.740	0.580 – 0.970	0.570 – 0.970
DAG(34:2)	0.600	0.590	0.450 – 0.860	0.440 – 0.840
PC (32:1)	0.770	0.760	0.670 – 0.920	0.660 – 0.950
PC (34:1)	0.400	0.410	0.210 – 0.850	0.200 – 0.830
PC (36:6)	0.690	0.670	0.540 – 0.940	0.510 – 0.930
PC (38:4)	0.600	0.610	0.410 – 0.930	0.420 – 0.910
PC (38:6)	0.450	0.440	0.270 – 0.750	0.250 – 0.710
LysoPC (15:0)	0.810	0.810	0.670 – 0.980	0.670 – 0.980

LysoPC (17:1)	0.590	0.630	0.390 – 0.930	0.430 – 0.940
LysoPC (18:0)	0.700	0.660	0.560 – 0.950	0.510 – 0.920
PC (34:4)	0.690	0.680	0.580 – 0.890	0.560 – 0.900
PC (36:2)	0.490	0.470	0.350 – 0.730	0.320 – 0.690
Hypoxanthine	0.760	0.750	0.560 – 0.970	0.550 – 0.970
PC (36:3)	0.550	0.540	0.360 – 0.920	0.350 – 0.910
PE (36:4)	0.770	0.760	0.690 – 0.880	0.680 – 0.880
PC (38:3)	0.620	0.610	0.490 – 0.750	0.470 – 0.770
LysoPC (22:6)	0.770	0.760	0.660 – 0.960	0.640 – 0.960
LysoPE (22:6)	0.710	0.700	0.600 – 0.920	0.570 – 0.920
Dimethylsulfone	0.770	0.770	0.650 – 0.950	0.650 – 0.950
LysoPC (16:1)	0.570	0.580	0.380 – 0.920	0.360 – 0.940
LysoPC (16:1)	0.820	0.830	0.740 – 0.960	0.730 – 0.980
LysoPC (20:2)	0.760	0.730	0.560 – 0.980	0.500 – 0.970
LysoPE (20:4)	0.790	0.800	0.680 – 0.970	0.680 – 0.970
LysoPC (14:0)	0.048	0.049	0.001 – 0.320	0.002 – 0.330
LysoPC (14:0)	0.700	0.720	0.540 – 0.950	0.540 – 0.970
Hexadecanoylcarnitine	0.700	0.710	0.590 – 0.910	0.600 – 0.930
LysoPC (15:1)	0.800	0.790	0.590 – 0.980	0.590 – 0.980
LysoPC (20:3)	0.630	0.610	0.500 – 0.840	0.490 – 0.810
LysoPC (18:1)	0.430	0.300	0.210 – 0.880	0.075 – 0.860
LysoPC (18:1)	0.880	0.860	0.750 – 0.990	0.710 – 0.990
ACar 13:1	0.610	0.610	0.490 – 0.780	0.480 – 0.800
Linoleylcarnitine	0.680	0.690	0.550 – 0.920	0.560 – 0.930
Plasmalogen PC (38:4) or PC (O-38:5)	0.700	0.710	0.600 – 0.860	0.600 – 0.860
LysoPC (20:6)	0.440	0.330	0.280 – 0.680	0.150 – 0.570
ACar 11:0	0.560	0.560	0.380 – 0.900	0.380 – 0.910
LysoPC (17:0)	0.810	0.760	0.680 – 1.000	0.630 – 0.960
DAG(36:4)	0.650	0.640	0.530 – 0.850	0.520 – 0.860
L,L-Cyclo(Ile-Pro)	0.820	0.820	0.750 – 0.890	0.740 – 0.920
Dodecadienylcarnitine	0.430	0.440	0.270 – 0.620	0.280 – 0.630
Hexadecatrienylcarnitine	0.600	0.600	0.470 – 0.740	0.470 – 0.770
L,L-Cyclo(Ile-Pro)	0.890	0.890	0.830 – 0.960	0.840 – 0.970
LysoPC (18:2)	0.800	0.720	0.680 – 0.980	0.560 – 0.960
LysoPC (18:2)	0.290	0.290	0.067 – 0.780	0.062 – 0.770
LysoPC (18:3)	0.510	0.470	0.300 – 0.780	0.240 – 0.770

LysoPC (18:3)	0.750	0.740	0.660 – 0.880	0.640 – 0.860
LysoPC (18:4)	0.840	0.840	0.770 – 0.960	0.760 – 0.970
LysoPC (18:4)	0.170	0.180	0.026 – 0.720	0.028 – 0.730
LysoPC (20:3)	0.880	0.880	0.760 – 0.990	0.750 – 0.990
LysoPC (20:3)	0.140	0.170	0.007 – 0.390	0.012 – 0.450
LysoPC (20:3)	0.130	0.140	0.005 – 0.430	0.006 – 0.500
LysoPC (20:3)	0.210	0.190	0.036 – 0.480	0.016 – 0.430
LysoPC (20:3)	0.580	0.580	0.440 – 0.790	0.440 – 0.790
LysoPC (20:4)	0.890	0.890	0.810 – 0.990	0.810 – 0.990
LysoPC (20:4)	0.860	0.840	0.780 – 0.980	0.750 – 0.980
LysoPC (20:5)	0.140	0.078	0.009 – 0.510	0.003 – 0.370
LysoPC (20:5)	0.840	0.790	0.770 – 0.960	0.710 – 0.910
LysoPC (22:5)	0.500	0.470	0.350 – 0.790	0.310 – 0.790
LysoPC (22:5)	0.450	0.430	0.220 – 0.810	0.190 – 0.800
LysoPE (16:0)	0.330	0.370	0.084 – 0.810	0.100 – 0.850
LysoPE (16:0)	0.530	0.530	0.370 – 0.860	0.350 – 0.880
LysoPE (18:2)	0.750	0.750	0.560 – 0.970	0.560 – 0.970
LysoPE (18:2)	0.440	0.350	0.160 – 0.880	0.096 – 0.830
Nonanoylcarnitine	0.850	0.850	0.730 – 0.980	0.720 – 0.980
Nonanoylcarnitine	0.610	0.660	0.420 – 0.860	0.460 – 0.900
Nonanoylcarnitine	0.660	0.630	0.500 – 0.930	0.470 – 0.910
PC (34:2)	0.580	0.570	0.450 – 0.740	0.440 – 0.710
PC (36:4)	0.630	0.630	0.510 – 0.760	0.520 – 0.760
PC (36:4)	0.770	0.770	0.690 – 0.890	0.690 – 0.890
PC (38:6)	0.500	0.500	0.350 – 0.740	0.350 – 0.760
PC (38:6)	0.780	0.770	0.660 – 0.970	0.660 – 0.970
PC (34:3)	0.540	0.540	0.290 – 0.900	0.310 – 0.910
Decenoylcarnitine	0.430	0.410	0.260 – 0.610	0.250 – 0.620
Decenoylcarnitine	0.480	0.470	0.330 – 0.700	0.320 – 0.670
PC (37:4)	0.750	0.750	0.590 – 0.940	0.580 – 0.940
PC (37:4)	0.630	0.630	0.420 – 0.920	0.410 – 0.920
PC (40:6)	0.740	0.730	0.610 – 0.950	0.600 – 0.970
PC (40:6)	0.480	0.440	0.300 – 0.770	0.250 – 0.730
PC (36:4)	0.610	0.520	0.460 – 0.880	0.370 – 0.810
PC (36:4)	0.860	0.860	0.800 – 0.930	0.800 – 0.940
PC (36:1)	0.860	0.880	0.780 – 0.940	0.810 – 0.950

PC (38:5)	0.720	0.720	0.630 – 0.820	0.630 – 0.830
PC (38:5)	0.700	0.680	0.560 – 0.930	0.540 – 0.920
Pipecolic acid	0.440	0.430	0.280 – 0.730	0.260 – 0.750
Ethyl glucoside	0.680	0.670	0.560 – 0.840	0.550 – 0.850
Serotonin	0.810	0.790	0.720 – 0.960	0.690 – 0.950
Serotonin	0.810	0.800	0.730 – 0.950	0.700 – 0.950
alpha-Tocopherol	0.910	0.910	0.880 – 0.950	0.870 – 0.950
Docosahexaenoic acid	0.640	0.620	0.500 – 0.900	0.490 – 0.870
Decadienylcarnitine	0.590	0.560	0.380 – 0.910	0.350 – 0.900
Decadienylcarnitine	0.660	0.650	0.470 – 0.930	0.450 – 0.930
Inosine	0.710	0.710	0.560 – 0.930	0.550 – 0.940
LysoPE (16:1)	0.630	0.660	0.450 – 0.920	0.460 – 0.940
LysoPE (16:1)	0.500	0.520	0.320 – 0.720	0.330 – 0.750
Propionylcarnitine	0.580	0.560	0.450 – 0.810	0.420 – 0.780
Trigonelline	0.860	0.850	0.730 – 0.980	0.710 – 0.980
Valine	0.400	0.330	0.240 – 0.590	0.160 – 0.530
Phenylalanine	0.550	0.530	0.350 – 0.930	0.330 – 0.910
trimethylamine N-oxide	0.190	0.140	0.031 – 0.670	0.016 – 0.610
Guanosine	0.670	0.670	0.530 – 0.920	0.530 – 0.930
Kynurenine	0.700	0.680	0.580 – 0.950	0.550 – 0.910
Tryptophan	0.440	0.410	0.250 – 0.860	0.210 – 0.850
Dodecenoylcarnitine	0.480	0.460	0.340 – 0.640	0.300 – 0.630
Dodecenoylcarnitine	0.430	0.450	0.260 – 0.700	0.270 – 0.790
Uric acid	0.180	0.180	0.016 – 0.540	0.013 – 0.490
Cortisol	0.760	0.770	0.610 – 0.970	0.610 – 0.970
Cortisone	0.670	0.660	0.510 – 0.950	0.490 – 0.950
Kynurenic acid	0.730	0.720	0.620 – 0.930	0.600 – 0.930
Tyrosine	0.230	0.210	0.045 – 0.530	0.025 – 0.520
Glycocholic acid	0.580	0.560	0.420 – 0.840	0.420 – 0.810
5-Acetylamino-6-amino-3-methyluracil	0.750	0.750	0.660 – 0.910	0.650 – 0.900
Leucine	0.570	0.550	0.430 – 0.810	0.420 – 0.800
Cholesterol	0.750	0.730	0.630 – 0.960	0.600 – 0.950
Isatin	0.740	0.750	0.600 – 0.950	0.600 – 0.950
Tetradecenoylcarnitine	0.650	0.650	0.530 – 0.800	0.530 – 0.830
Tetradecenoylcarnitine	0.560	0.580	0.390 – 0.730	0.400 – 0.760
Glycochenodeoxycholic acid	0.670	0.670	0.520 – 0.920	0.510 – 0.920

Glycodeoxycholic acid	0.480	0.460	0.320 – 0.650	0.300 – 0.650
Leu-Val	0.460	0.450	0.230 – 0.890	0.210 – 0.900
Trp-Phe	0.840	0.850	0.700 – 0.980	0.700 – 0.980
Creatine	0.830	0.810	0.740 – 0.960	0.710 – 0.960
gamma-CEHC	0.600	0.620	0.440 – 0.910	0.430 – 0.940
Sphingosine	0.780	0.780	0.670 – 0.900	0.670 – 0.910
Retinol	0.720	0.740	0.560 – 0.960	0.570 – 0.970
Indole-3-acetic acid	0.460	0.460	0.310 – 0.650	0.310 – 0.680
Proline	0.580	0.570	0.430 – 0.880	0.420 – 0.860
Tetradecadienylcarnitine	0.490	0.500	0.320 – 0.760	0.330 – 0.770
Tetradecadienylcarnitine	0.310	0.330	0.120 – 0.540	0.130 – 0.610
Caffeine	0.850	0.850	0.790 – 0.900	0.790 – 0.910
Theobromine	0.220	0.210	0.036 – 0.460	0.025 – 0.460
Theophylline	0.610	0.600	0.480 – 0.730	0.470 – 0.730
Phenylacetylglutamine	0.730	0.740	0.610 – 0.930	0.620 – 0.930
2-Hydroxy-3-methylbutyric acid	0.870	0.870	0.820 – 0.930	0.810 – 0.940
ACar 11:1	0.790	0.780	0.610 – 0.970	0.600 – 0.970
ACar 13:0	0.700	0.690	0.590 – 0.890	0.570 – 0.890
O-Palmitoleoylcarnitine	0.690	0.690	0.560 – 0.940	0.550 – 0.940
Isoleucine	0.600	0.600	0.460 – 0.890	0.450 – 0.890
Citrulline	0.440	0.430	0.290 – 0.690	0.280 – 0.690
Butyrylcarnitine	0.720	0.730	0.620 – 0.850	0.630 – 0.870
Butyrylcarnitine	0.640	0.600	0.510 – 0.810	0.450 – 0.850
Methionine	0.210	0.180	0.035 – 0.560	0.020 – 0.590
Glycoursodeoxycholic acid	0.800	0.810	0.680 – 0.940	0.690 – 0.940
Asp-Phe	0.730	0.730	0.440 – 0.960	0.420 – 0.950
Phe-Phe	0.750	0.750	0.570 – 0.960	0.560 – 0.960
Decanoylcarnitine	0.590	0.580	0.450 – 0.770	0.450 – 0.800
Octanoylcarnitine	0.600	0.580	0.470 – 0.780	0.440 – 0.760
Hexadecenoylcarnitine	0.630	0.610	0.520 – 0.760	0.480 – 0.830
Hexanoylcarnitine	0.620	0.590	0.490 – 0.770	0.450 – 0.820
SM d32:2	0.640	0.620	0.510 – 0.780	0.490 – 0.820
SM d33:1	0.620	0.620	0.470 – 0.900	0.460 – 0.910
Valerylcarnitine	0.330	0.320	0.160 – 0.530	0.150 – 0.550
Valerylcarnitine	0.270	0.250	0.050 – 0.860	0.033 – 0.610
SM d34:1	0.540	0.540	0.370 – 0.860	0.380 – 0.850

SM d34:2	0.790	0.780	0.710 – 0.920	0.690 – 0.920
SM d36:2	0.500	0.490	0.350 – 0.710	0.330 – 0.740
SM d32:1	0.830	0.820	0.750 – 0.990	0.730 – 0.960
N1-Acetylspermidine	0.610	0.620	0.500 – 0.760	0.500 – 0.790
Acetylcarnitine	0.530	0.520	0.390 – 0.700	0.370 – 0.750
Tetradecanoylcarnitine	0.640	0.640	0.500 – 0.910	0.480 – 0.930
Dodecanoylcarnitine	0.480	0.470	0.330 – 0.740	0.310 – 0.770
Paraxanthine	0.860	0.860	0.800 – 0.910	0.800 – 0.920
Bilirubin isomer 1	0.690	0.690	0.560 – 0.910	0.570 – 0.900
Oleoylcarnitine	0.910	0.900	0.780 – 0.990	0.760 – 0.980
Oleoylcarnitine	0.260	0.260	0.079 – 0.540	0.070 – 0.540

Figure S1: distribution of proportion of samples where compound was present.

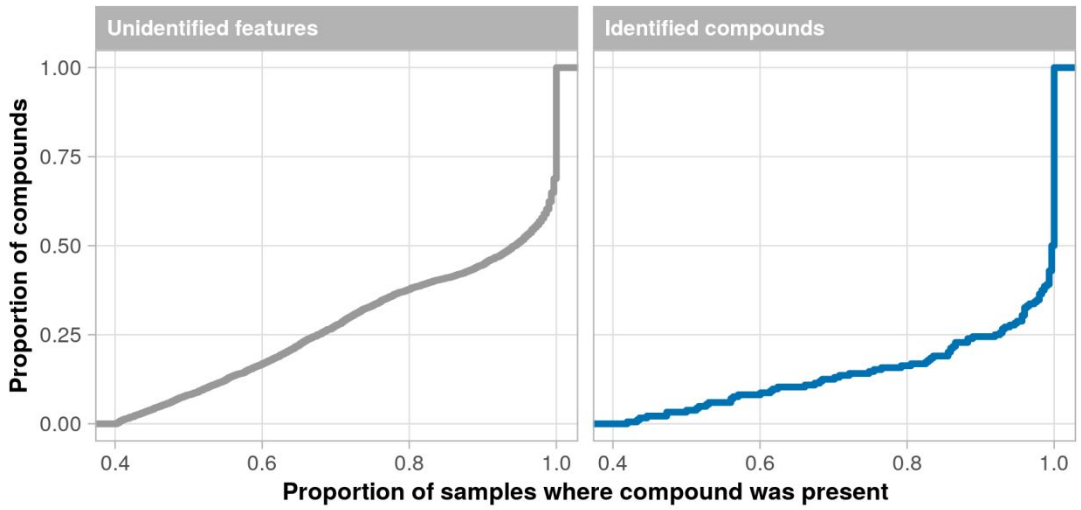

Figure S2: further decomposition of the components of the ICC calculation from the unadjusted, three-level tobit model of the paper (equation 1 paper). $\boldsymbol{\sigma_1^2}$ is the modelled within-subject variance, $\boldsymbol{\sigma_2^2}$ the between-subject variance, and $\boldsymbol{\sigma_3^2}$ describes the between-centre variance. ICC = intraclass correlation coefficient. 'Identified' refers to if the feature could be annotated.

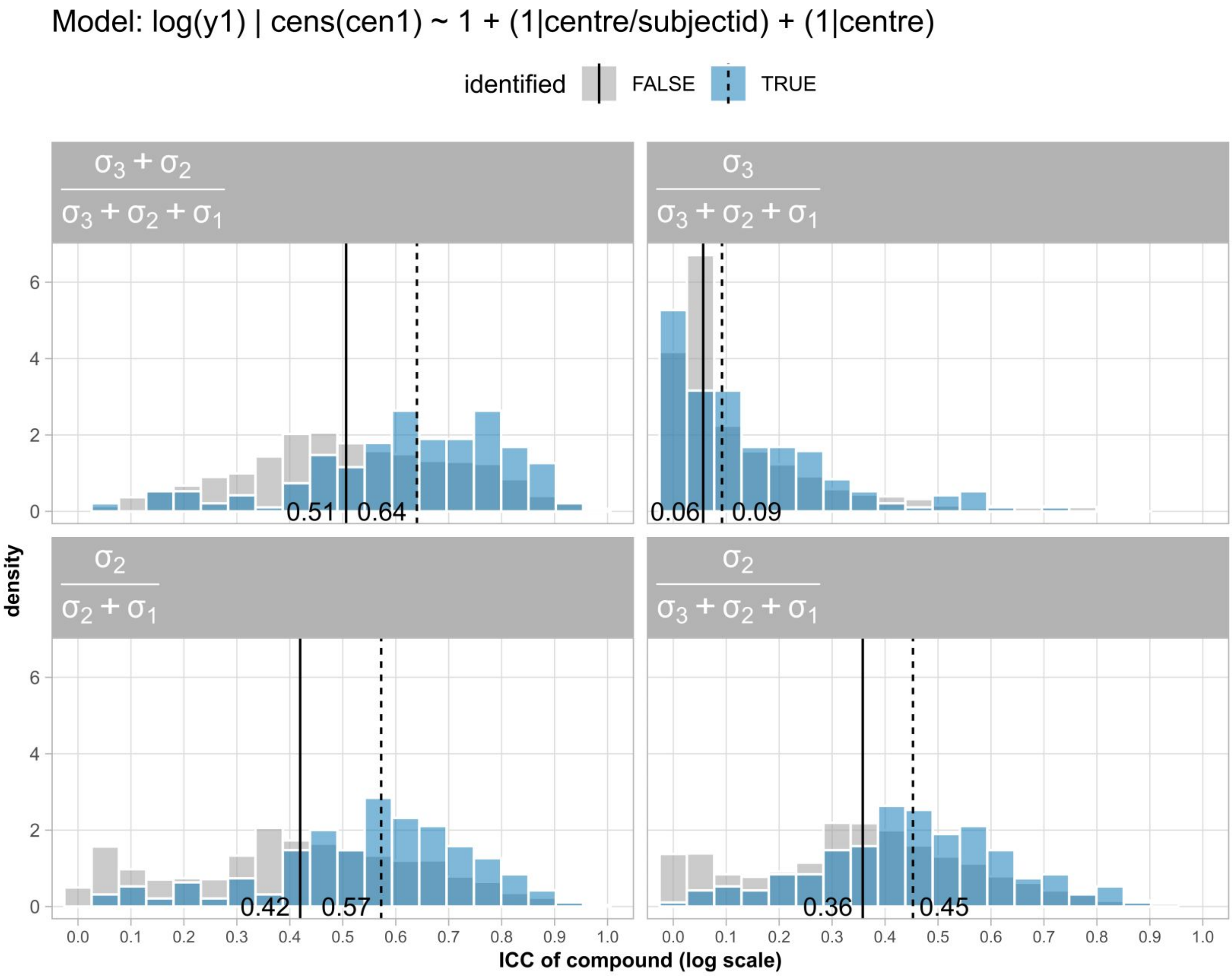

Figure S3: proportion of variance attributable to between-subject, between-center and within-subject variation for all uniquely identified compounds according to the unadjusted, three-level tobit model of the paper (equation 1 paper).

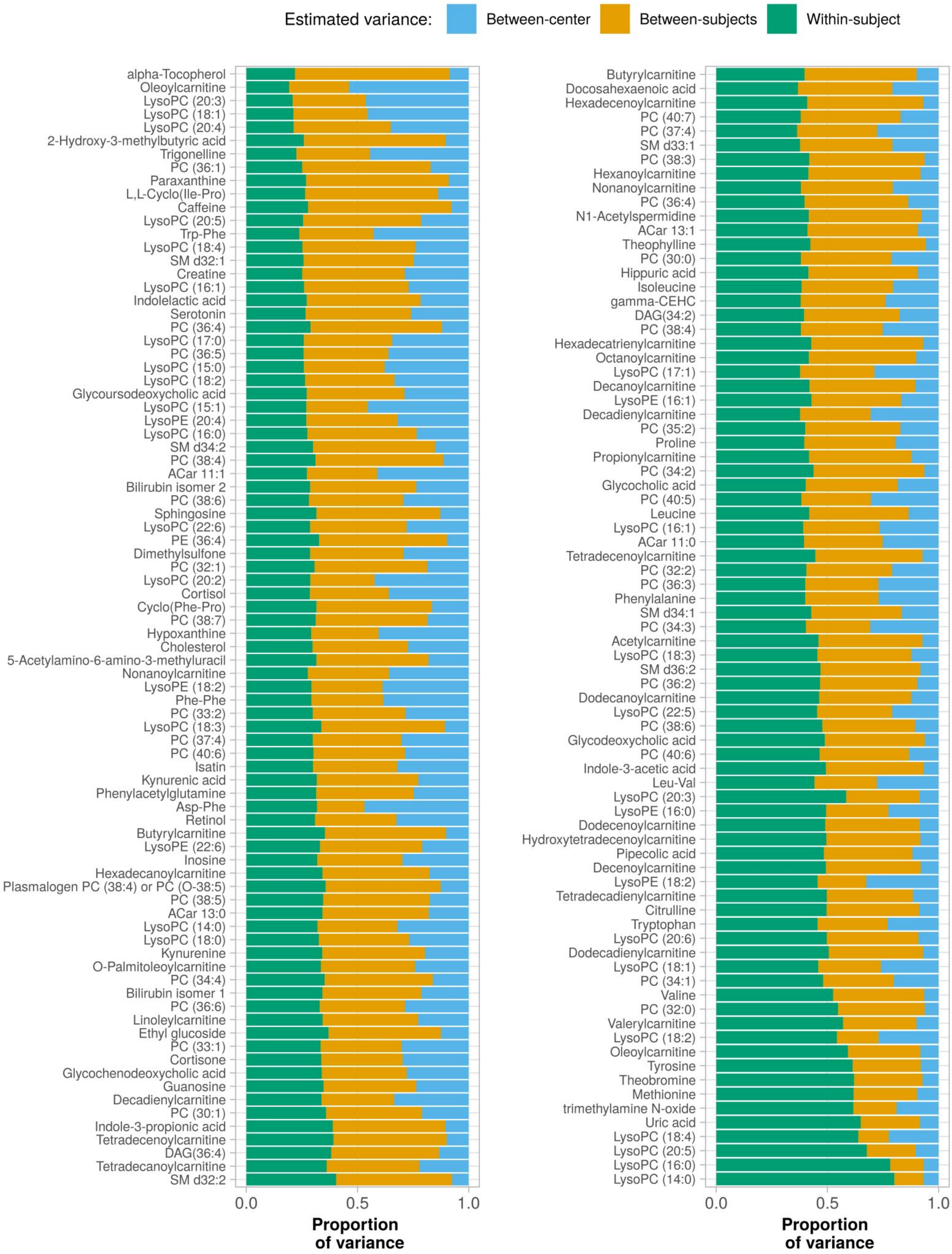

Figure S4: distribution of ICCs according to the adjusted model where we have added fixed effects for age, BMI and sex to the three-level tobit model of the paper (equation 1 paper). Age was a smooth term with brms defaults. $\boldsymbol{\sigma_1^2}$ is the modelled within-subject variance, $\boldsymbol{\sigma_2^2}$ the between-subject variance, and $\boldsymbol{\sigma_3^2}$ describes the between-centre variance. ICC = intraclass correlation coefficient.

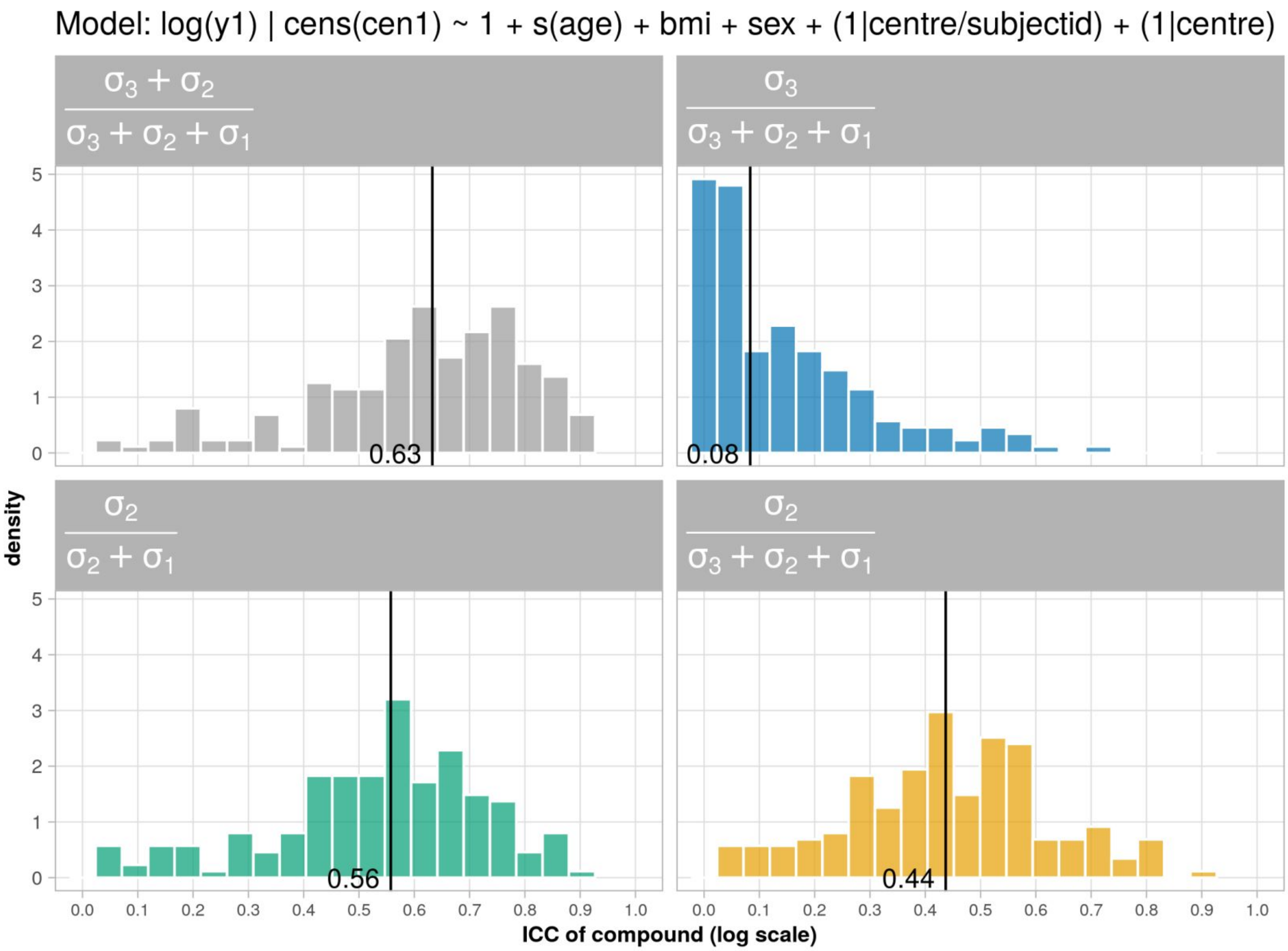

Figure S5: distribution of ICCs grouped by chemical class and biological pathway. ICCs based on the adjusted model where we have added fixed effects for age, BMI and sex to the three-level tobit model of the paper (equation 1 paper). Age was a smooth term with brms defaults. Only pathways with at least four entries are shown. The transparent dots in the boxplot are jitter and show all individual data points. ICC = intraclass correlation coefficient.

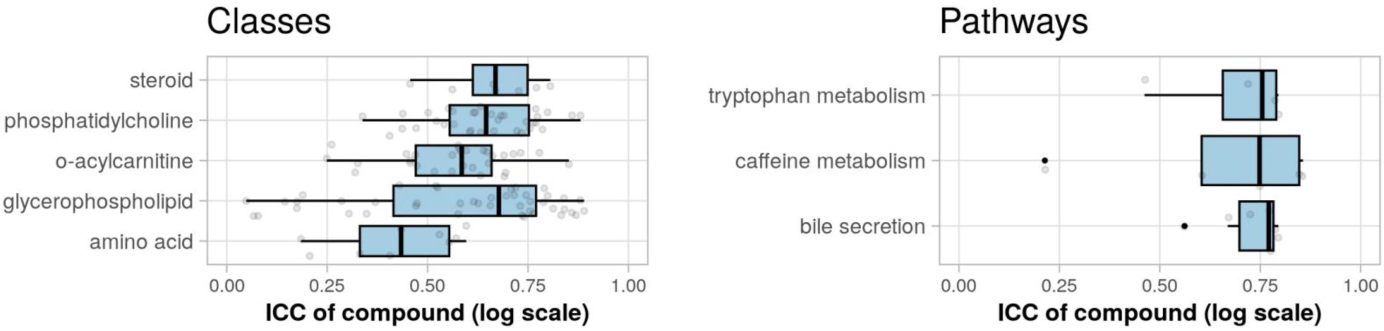

Figure S6: distribution of ICCs by their identification status with the unadjusted two-level model from Supplementary Methods 1. Details on the conversion from log scale to data scale can be found in Supplementary Methods 2. ICC = intraclass correlation coefficient. ‘Identified’ refers to if the feature could be annotated.

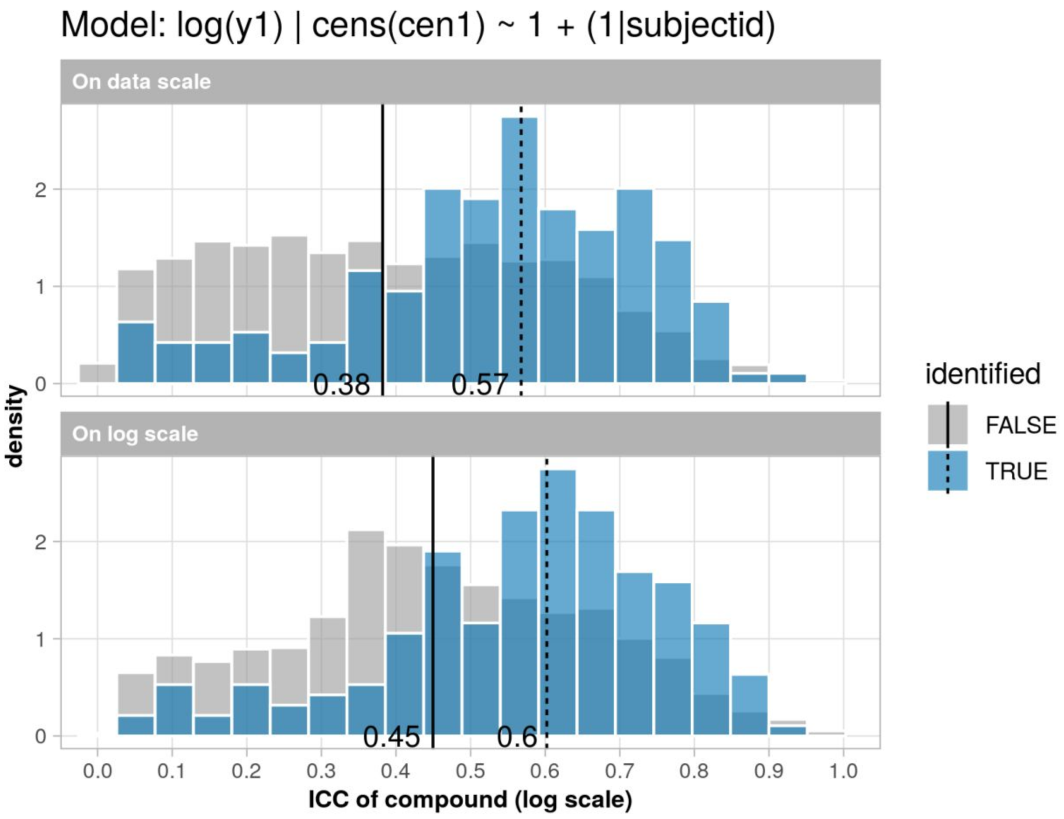

Figure S7: distribution of ICCs stratified by centre with the unadjusted two-level model from Supplementary Methods 1 (identified compounds only). The vertical line indicates the median value per centre. ICC = intraclass correlation coefficient.

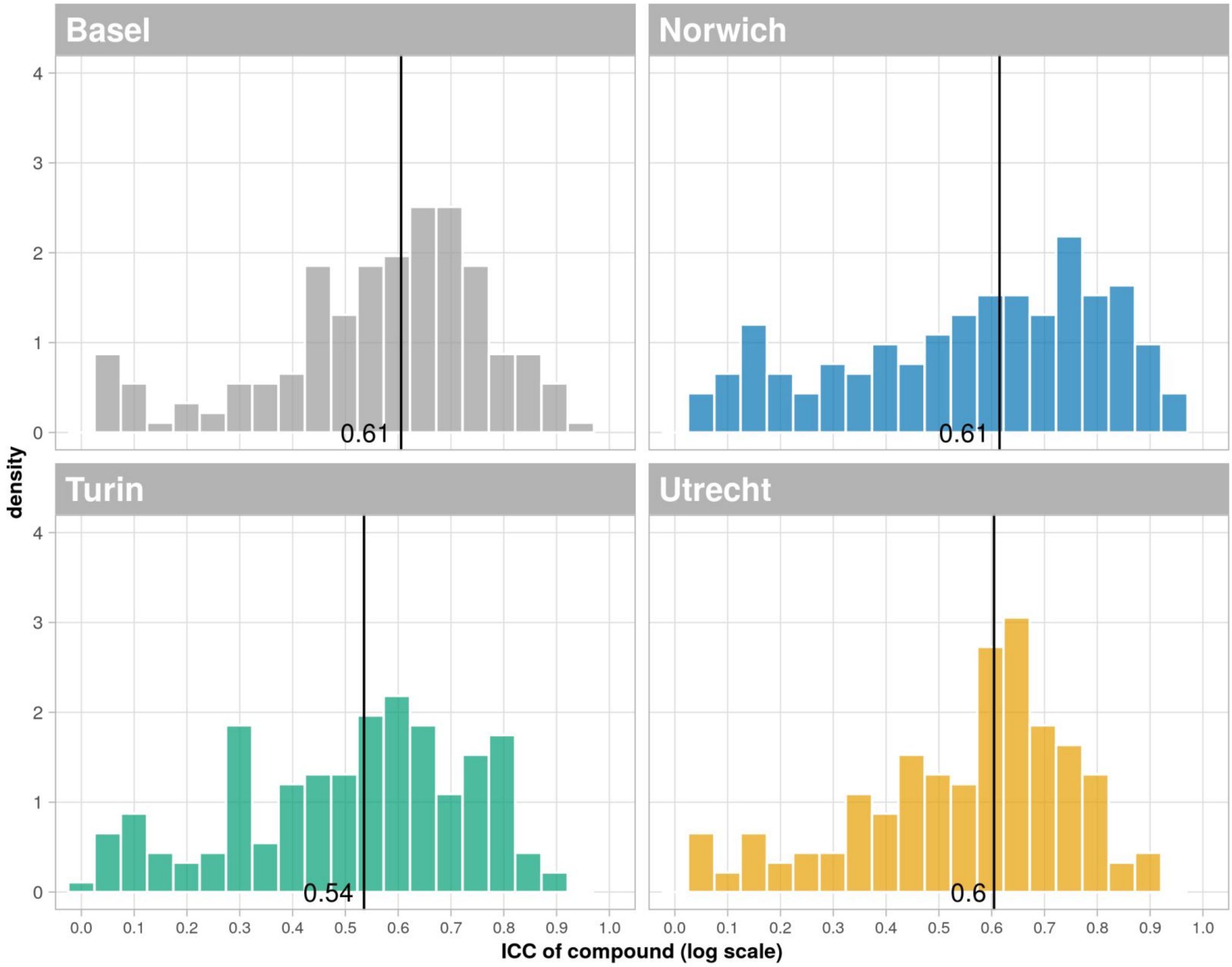

Figure S8: comparison to ICCs reported based on targeted assays. The colored line indicates the overall median ICC per study for all compounds that were matched. The dot indicates the estimated ICC of a compound by a study, and the line that goes through the dot is the 95% confidence interval or credible interval. ICC = intraclass correlation coefficient. Floegel (2011) [4], Yin (2022) [5], Breier (2014) [6].

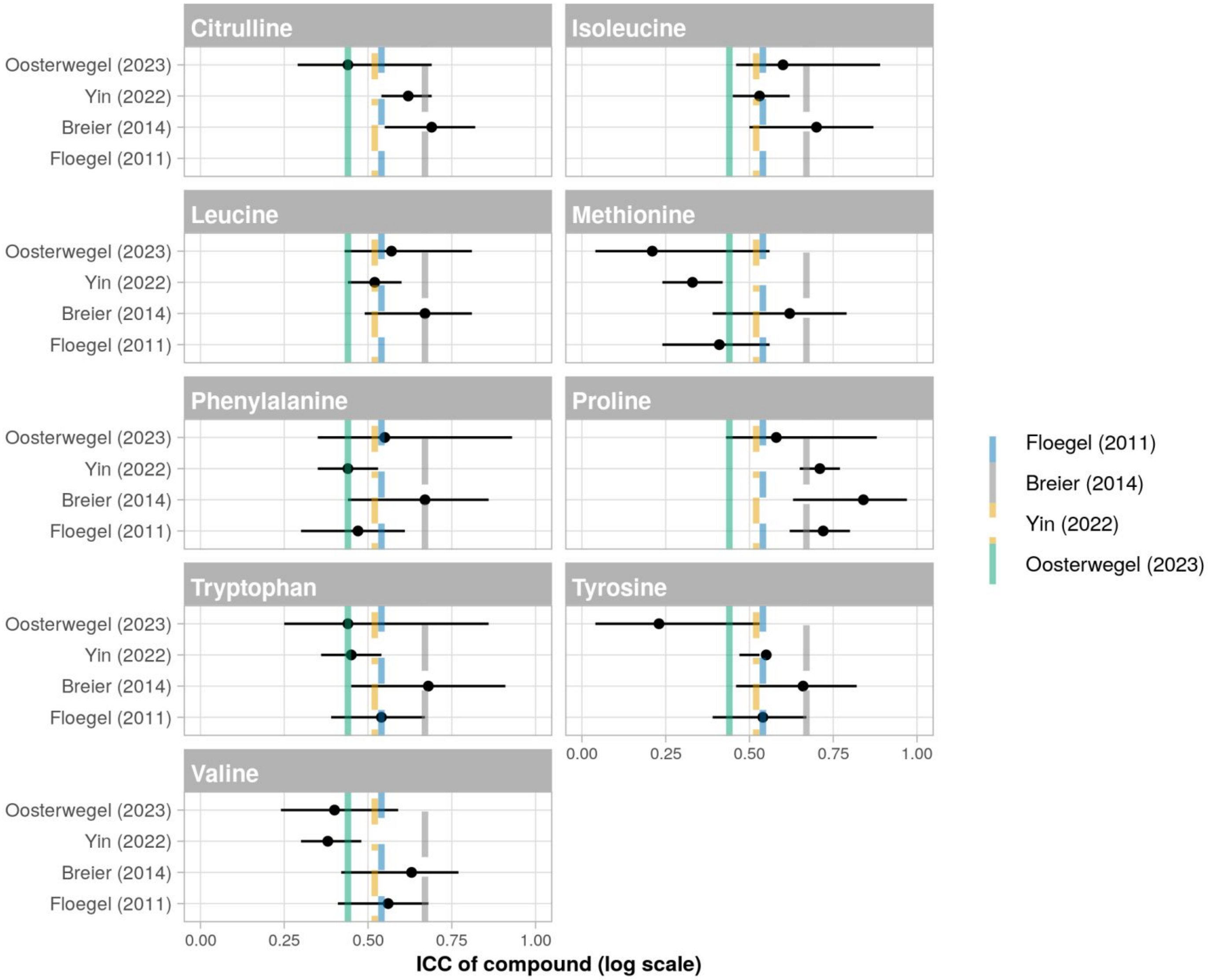

References

(1) de Villemereuil, P.; Schielzeth, H.; Nakagawa, S.; Morrissey, M. General Methods for Evolutionary Quantitative Genetic Inference from Generalized Mixed Models. *Genetics* **2016**, *204* (3), 1281–1294. https://doi.org/10.1534/genetics.115.186536.

(2) Magnusson, K. *Estimating treatment effects and ICCs from (G)LMMs on the observed scale using Bayes, Part 1: lognormal models*. https://rpsychologist.com/GLMM-part1-lognormal (accessed 2022-11-09).

(3) Sumner, L. W.; Amberg, A.; Barrett, D.; Beale, M. H.; Beger, R.; Daykin, C. A.; Fan, T. W.-M.; Fiehn, O.; Goodacre, R.; Griffin, J. L.; Hankemeier, T.; Hardy, N.; Harnly, J.; Higashi, R.; Kopka, J.; Lane, A. N.; Lindon, J. C.; Marriott, P.; Nicholls, A. W.; Reily, M. D.; Thaden, J. J.; Viant, M. R. Proposed Minimum Reporting Standards for Chemical Analysis Chemical Analysis Working Group (CAWG) Metabolomics Standards Initiative (MSI). *Metabolomics* **2007**, *3* (3), 211–221. https://doi.org/10.1007/s11306-007-0082-2.

(4) Floegel, A.; Drogan, D.; Wang-Sattler, R.; Prehn, C.; Illig, T.; Adamski, J.; Joost, H.-G.; Boeing, H.; Pischon, T. Reliability of Serum Metabolite Concentrations over a 4-Month Period Using a Targeted Metabolomic Approach. *PLOS ONE* **2011**, *6* (6), e21103. https://doi.org/10.1371/journal.pone.0021103.

(5) Yin, X.; Prendiville, O.; McNamara, A. E.; Brennan, L. Targeted Metabolomic Approach to Assess the Reproducibility of Plasma Metabolites over a Four Month Period in a Free-Living Population. *J. Proteome Res.* **2022**, *21* (3), 683–690. https://doi.org/10.1021/acs.jproteome.1c00440.

(6) Breier, M.; Wahl, S.; Prehn, C.; Fugmann, M.; Ferrari, U.; Weise, M.; Banning, F.; Seissler, J.; Grallert, H.; Adamski, J.; Lechner, A. Targeted Metabolomics Identifies Reliable and Stable Metabolites in Human Serum and Plasma Samples. *PLoS One* **2014**, *9* (2), e89728. https://doi.org/10.1371/journal.pone.0089728.
